# Supplementary figures and images for: Validation of Friedewald, Martin-Hopkins and Sampson low-density lipoprotein cholesterol equations
Source: PLoS One. 2022 May 13;17(5):e0263860. doi: 10.1371/journal.pone.0263860 (PMC9106156; doi:10.1371/journal.pone.0263860)

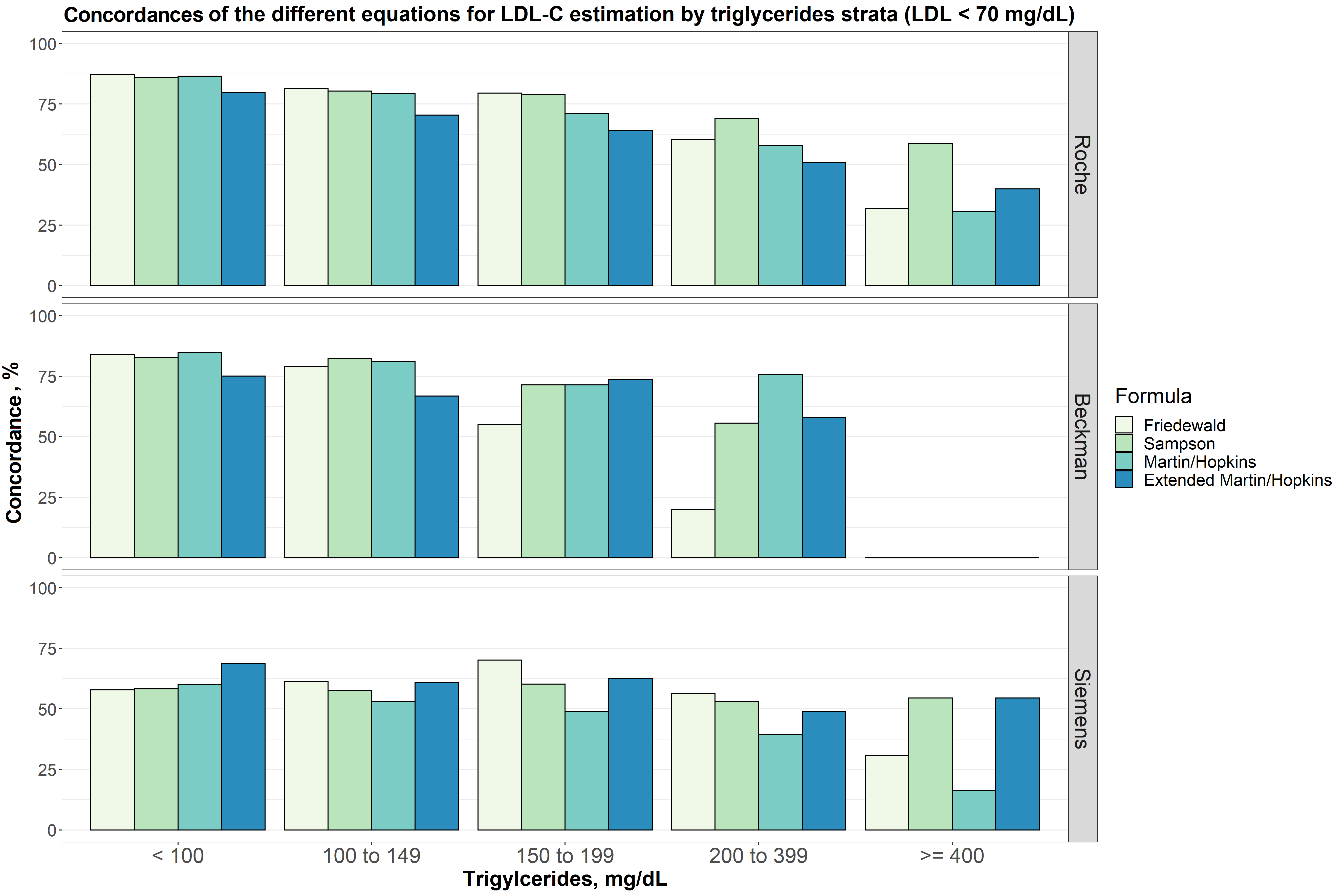

Supplement: S1 Fig — (PNG) [file pone.0263860.s001.png]

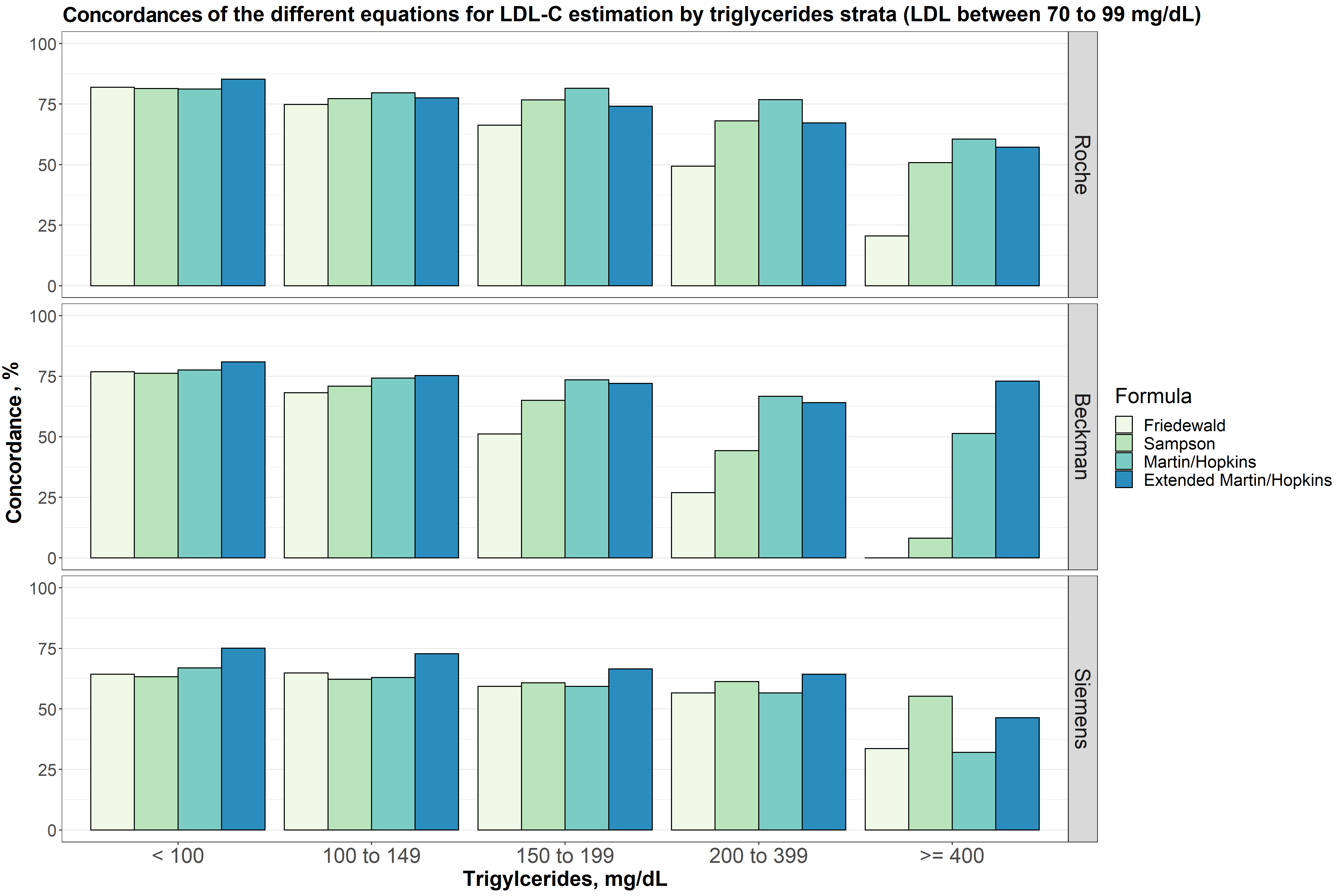

Supplement: S2 Fig — (PNG) [file pone.0263860.s002.png]

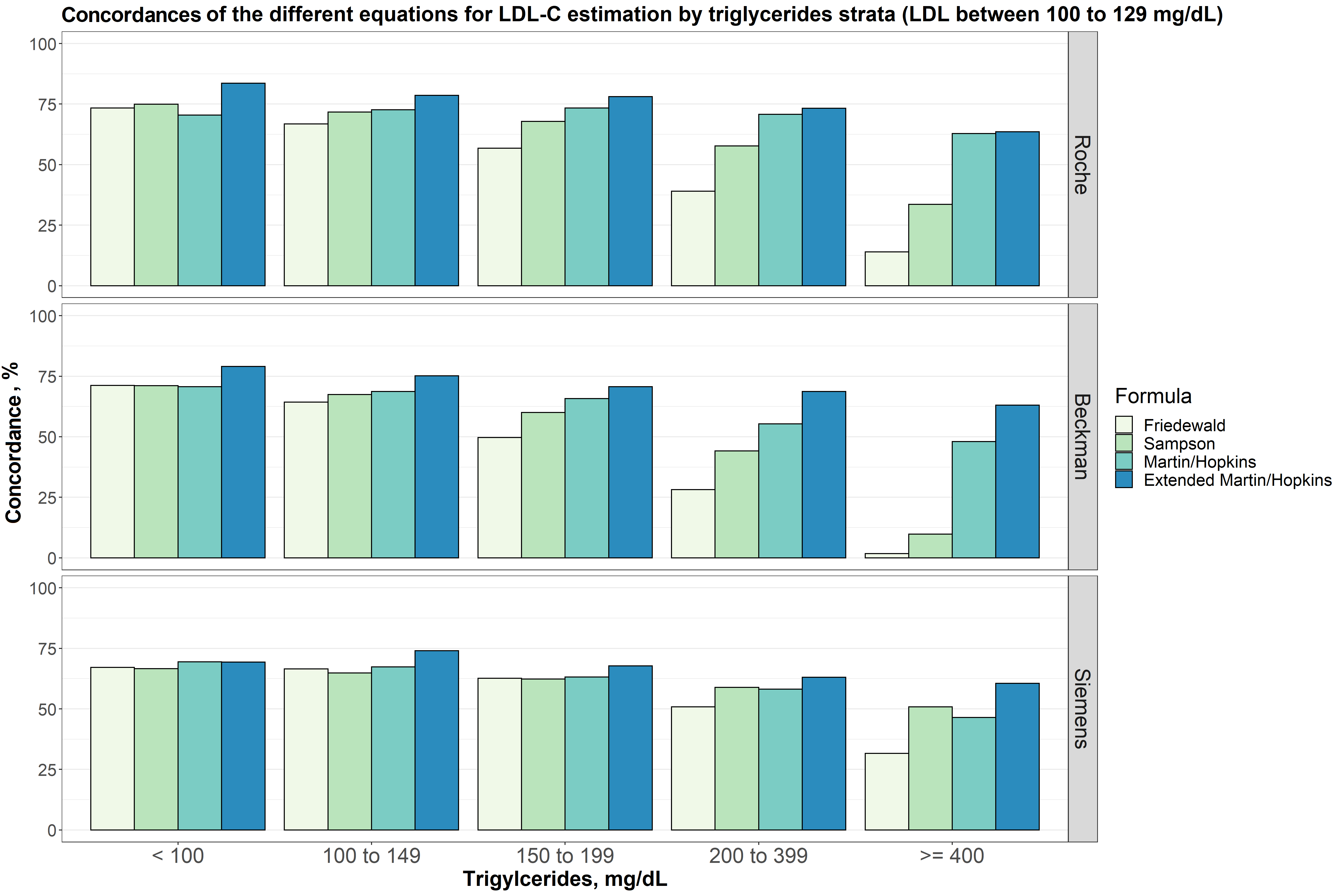

Supplement: S3 Fig — (PNG) [file pone.0263860.s003.png]

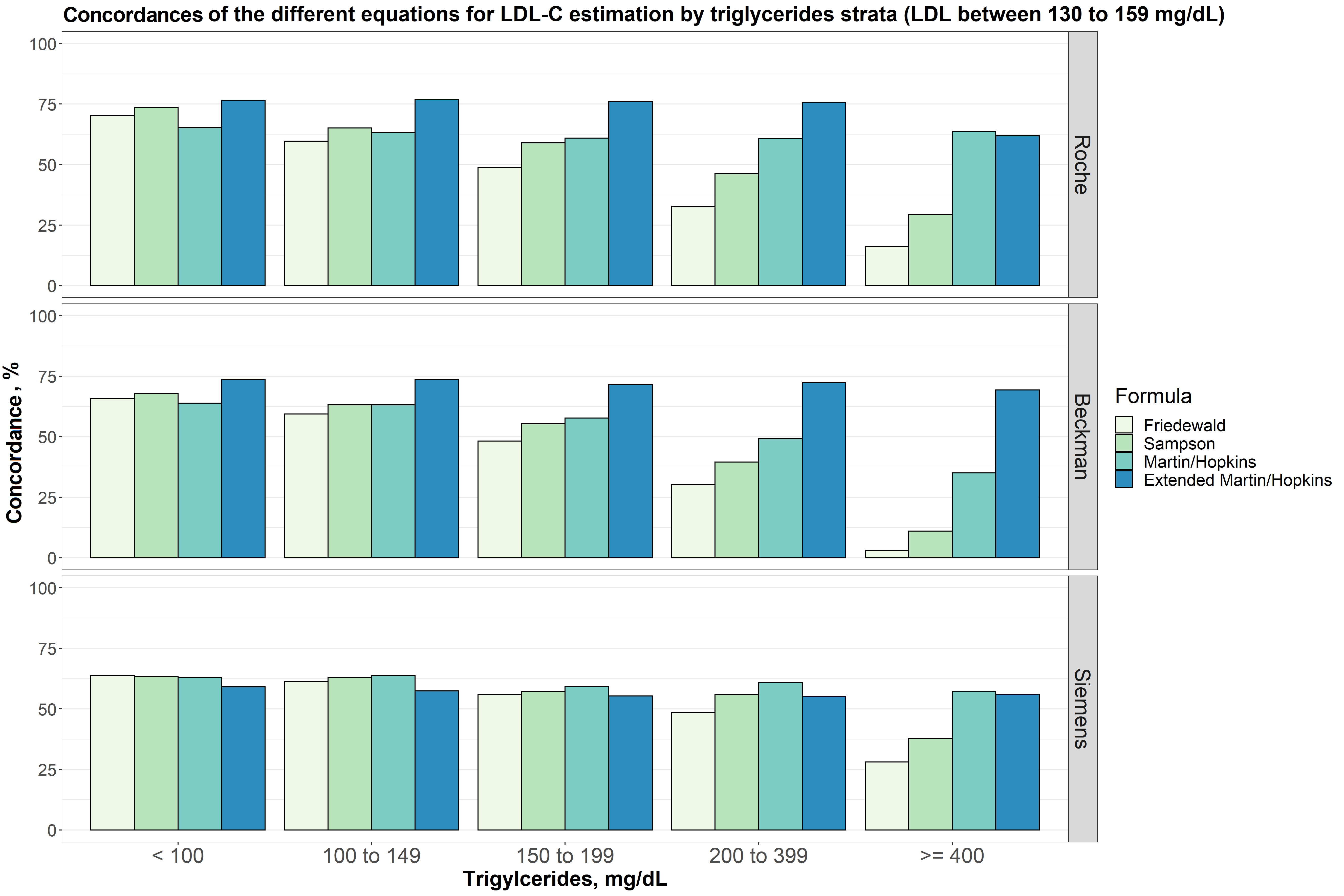

Supplement: S4 Fig — (PNG) [file pone.0263860.s004.png]

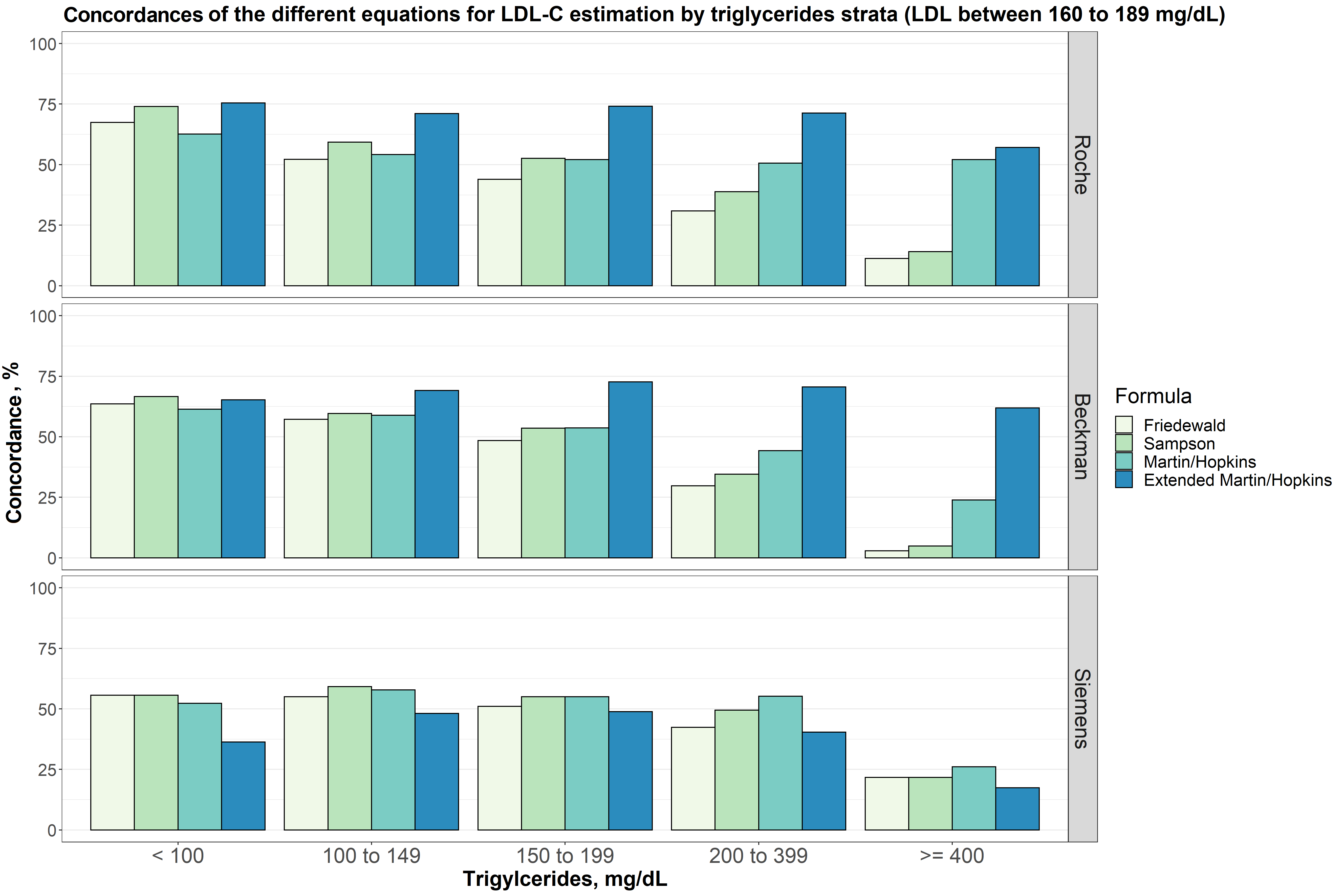

Supplement: S5 Fig — (PNG) [file pone.0263860.s005.png]

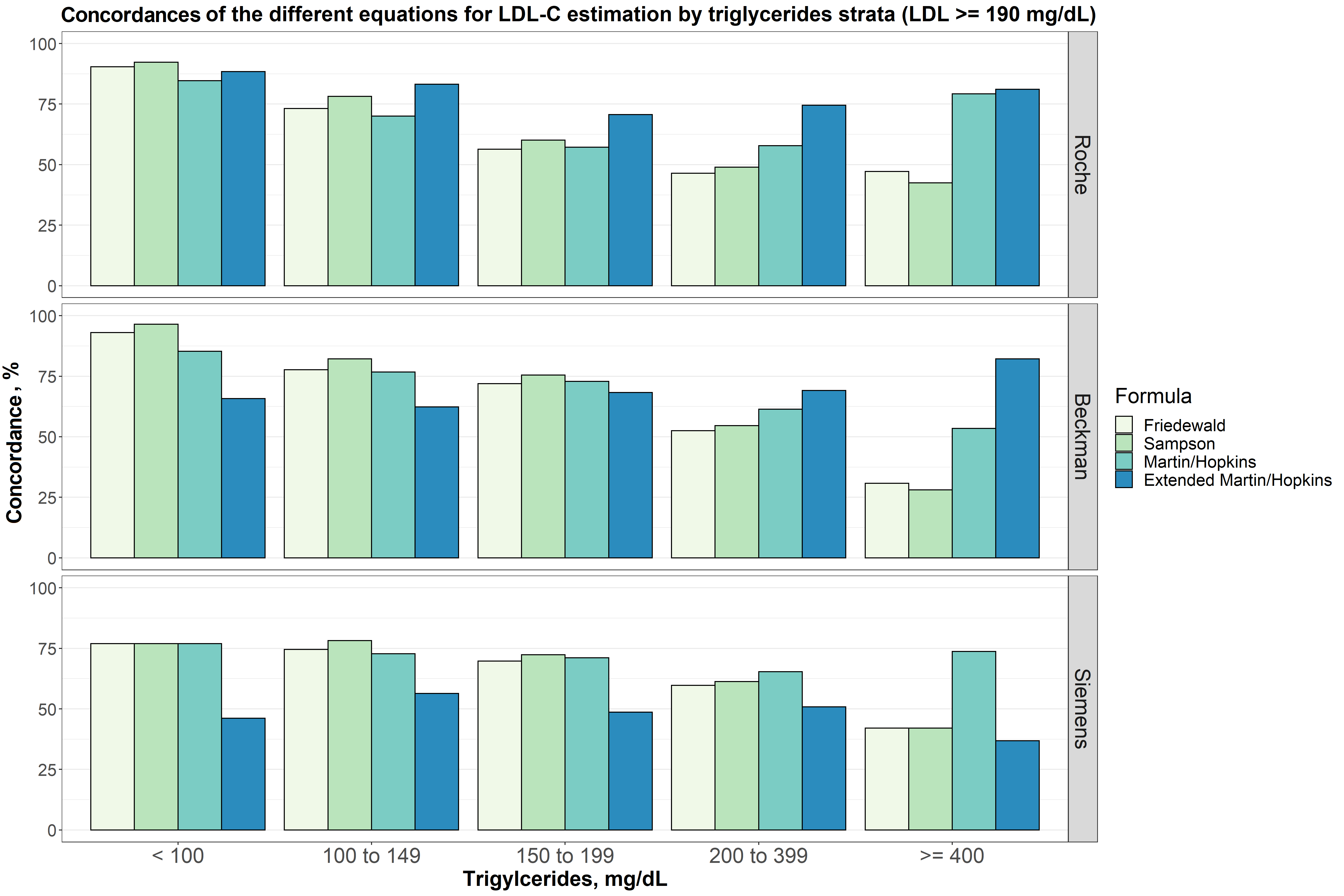

Supplement: S6 Fig — (PNG) [file pone.0263860.s006.png]

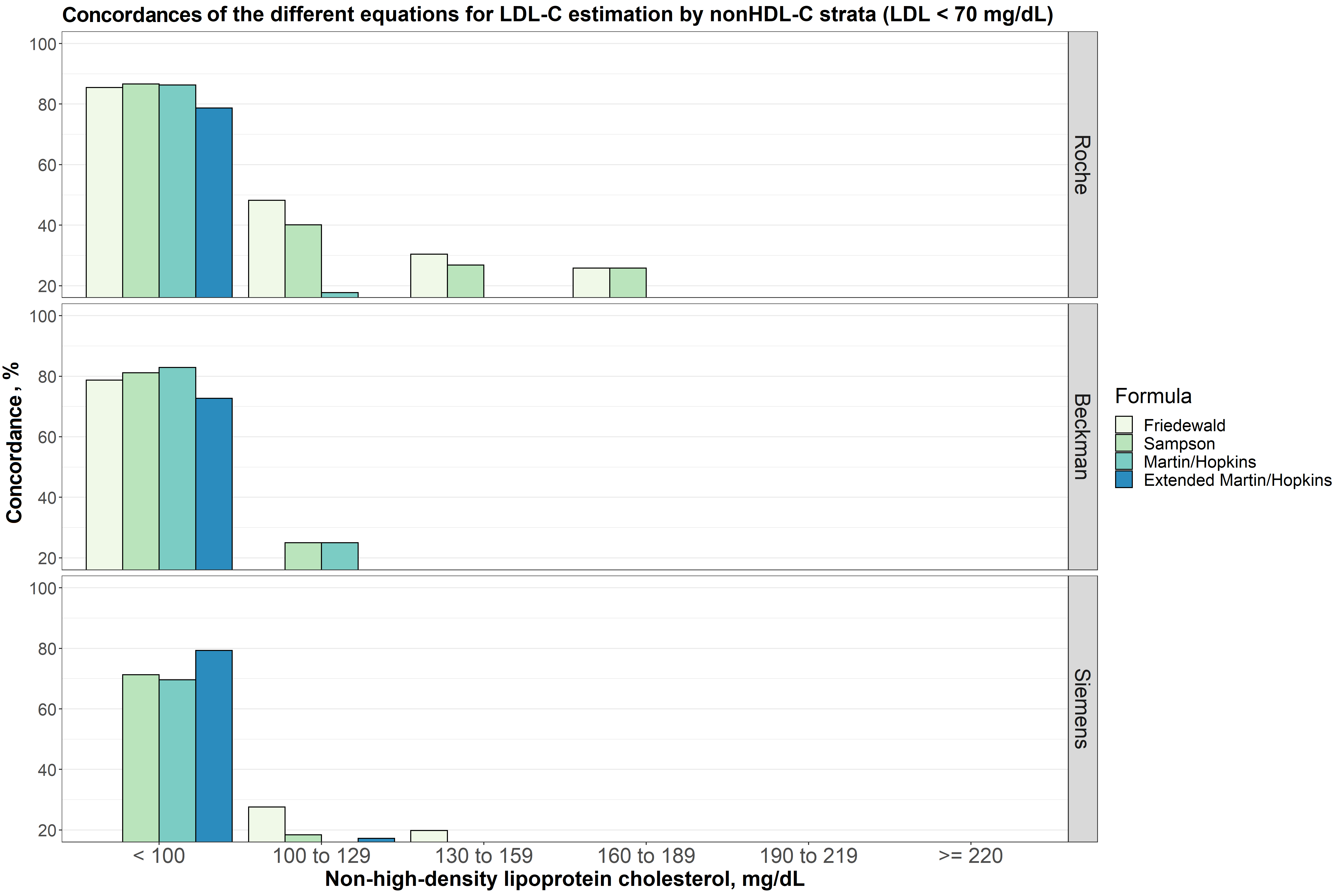

Supplement: S7 Fig — (PNG) [file pone.0263860.s007.png]

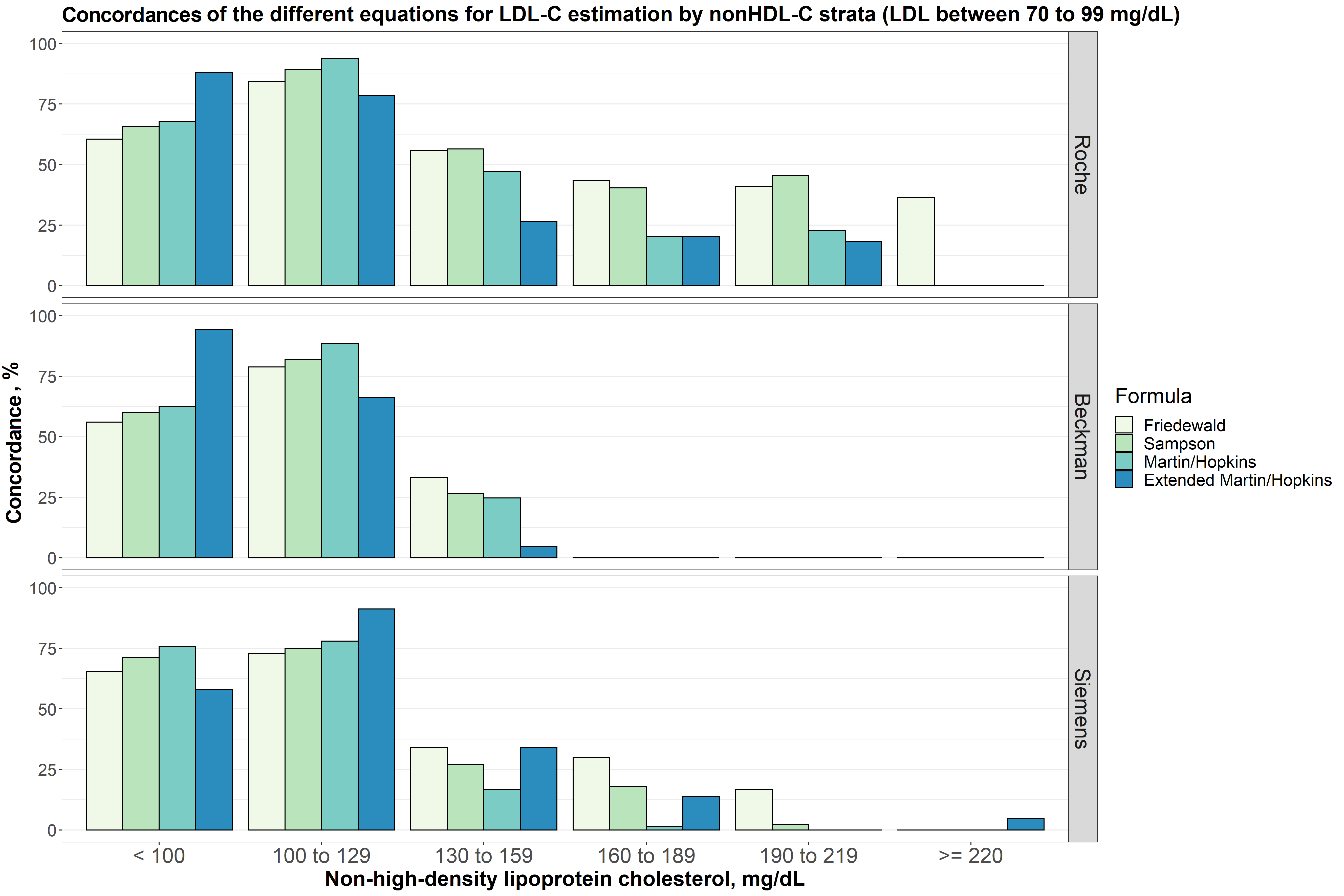

Supplement: S8 Fig — (PNG) [file pone.0263860.s008.png]

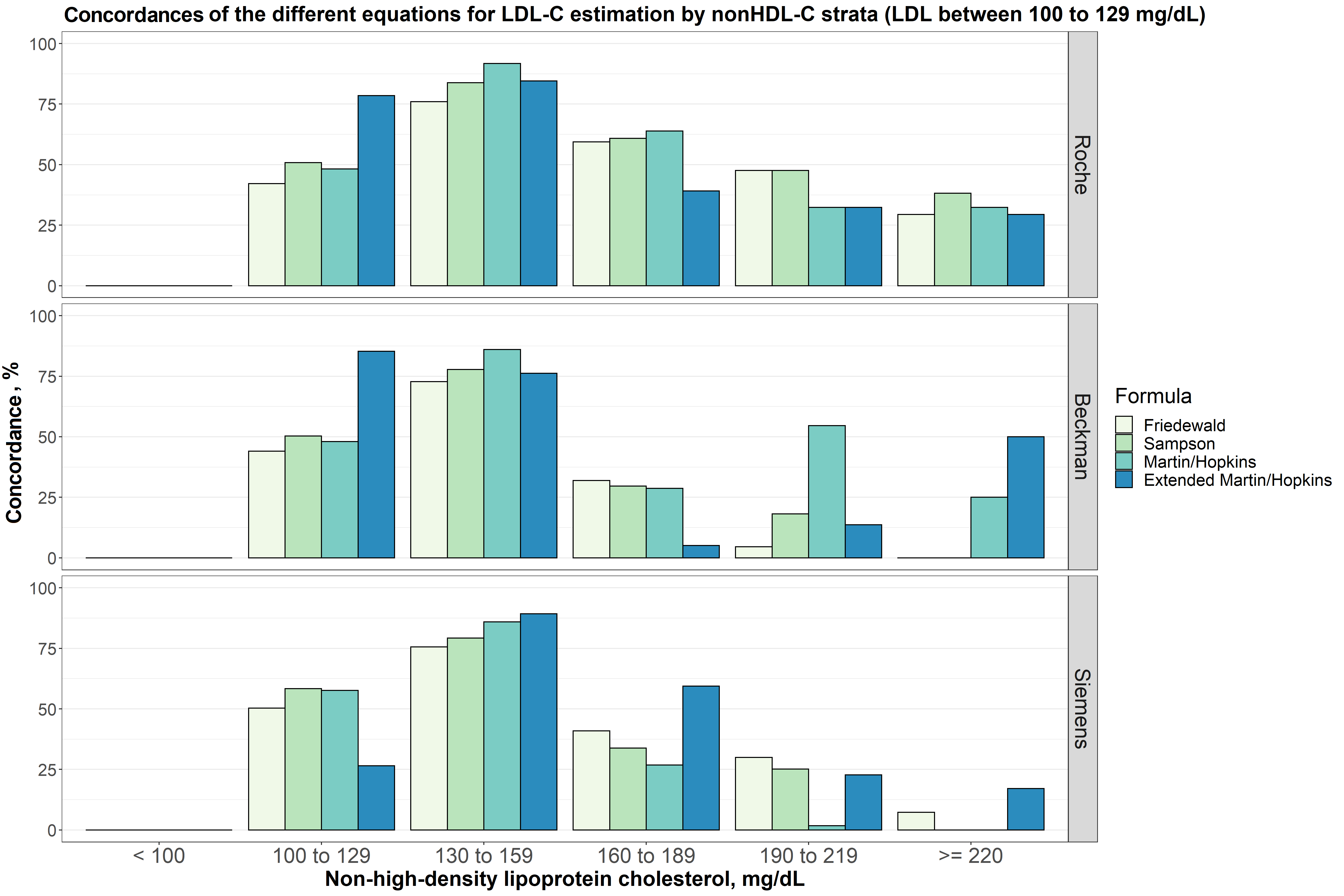

Supplement: S9 Fig — (PNG) [file pone.0263860.s009.png]

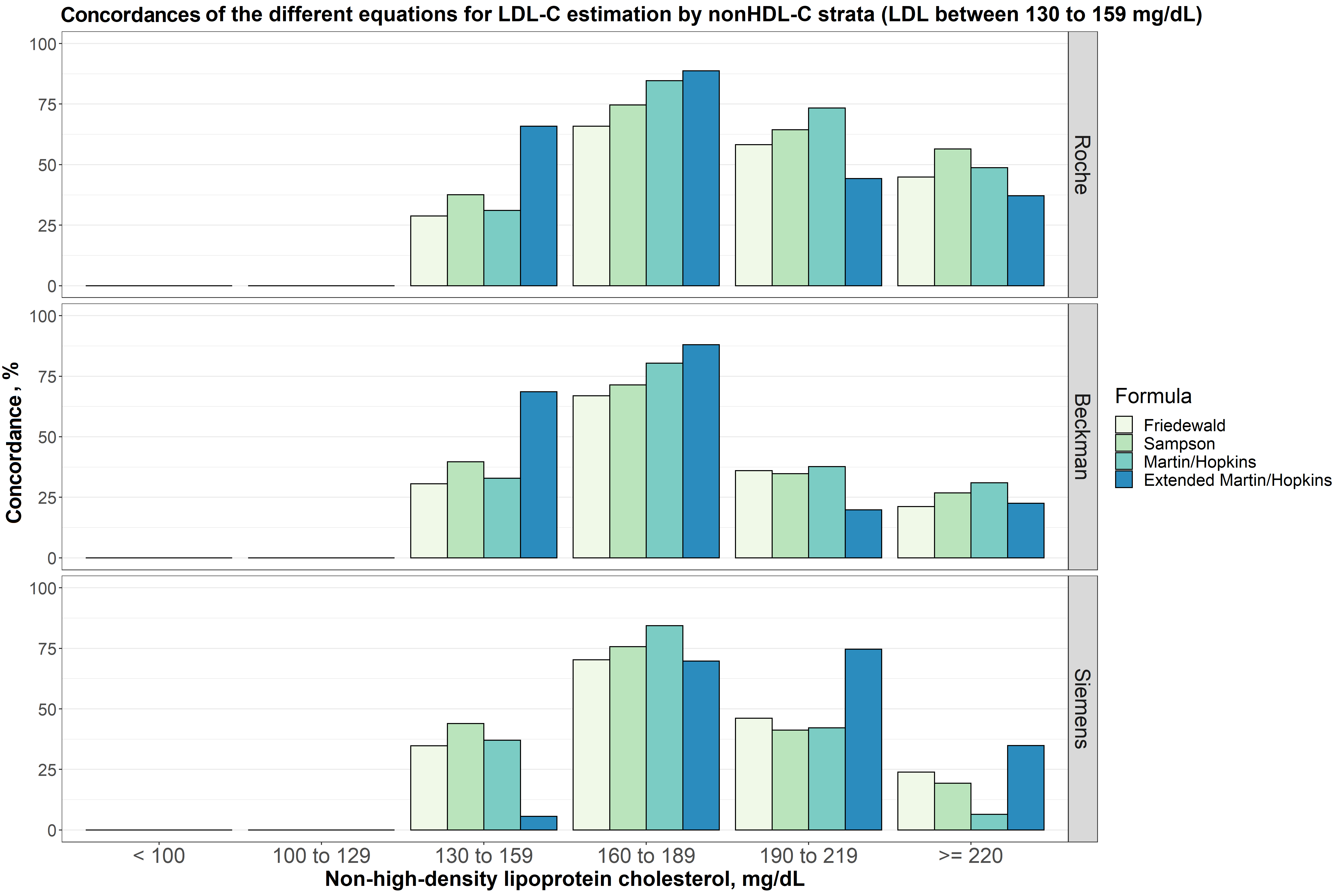

Supplement: S10 Fig — (PNG) [file pone.0263860.s010.png]

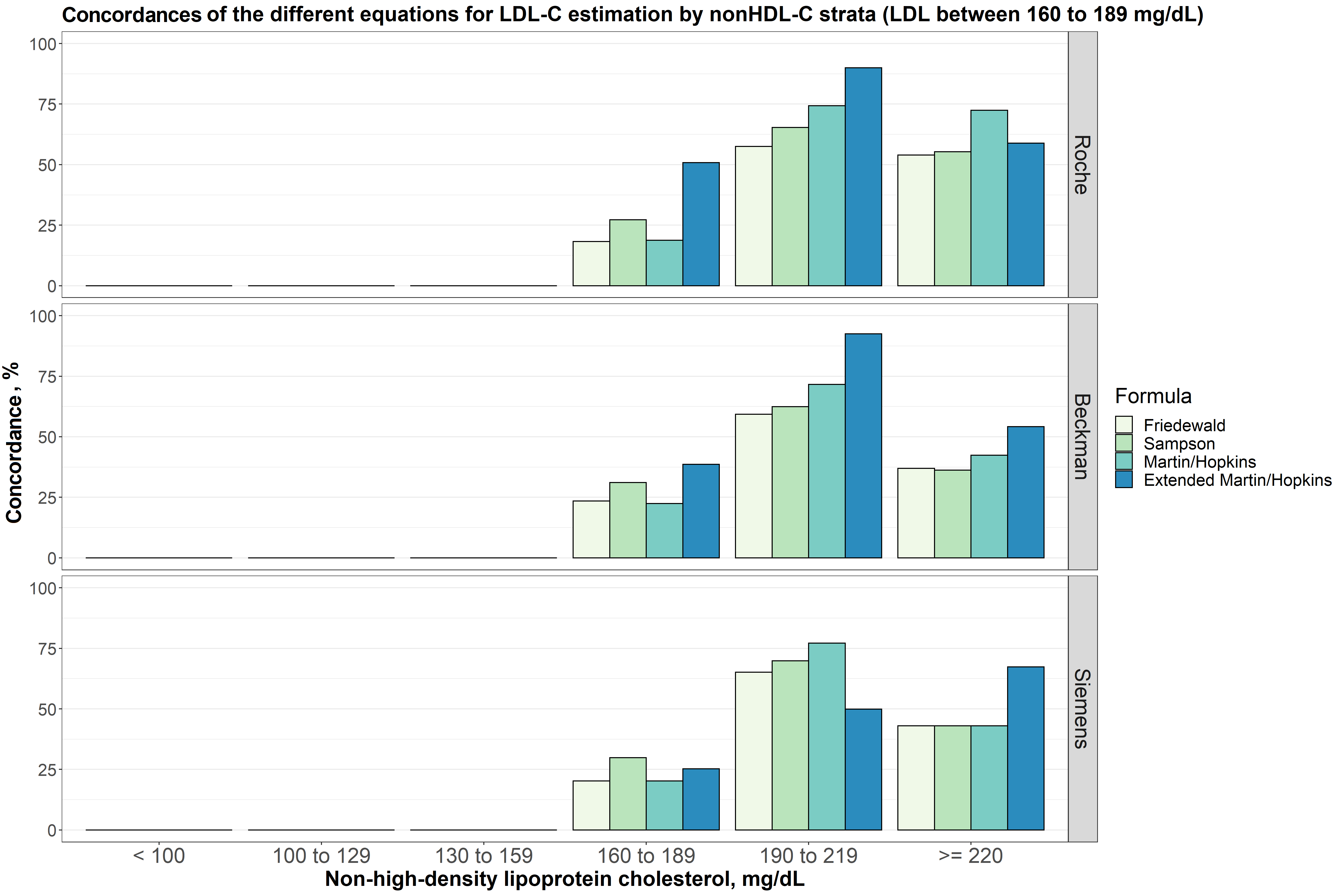

Supplement: S11 Fig — (PNG) [file pone.0263860.s011.png]

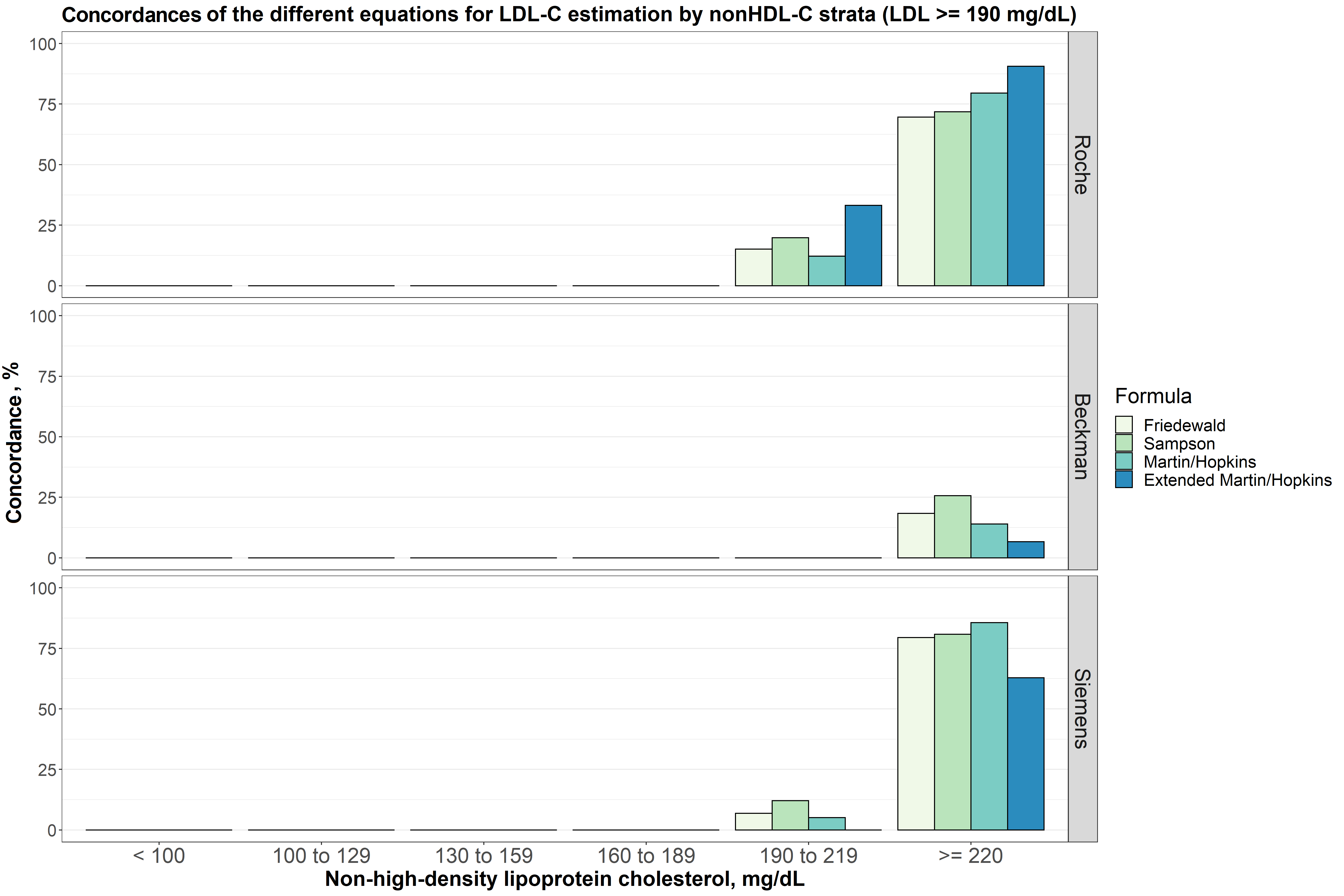

Supplement: S12 Fig — (PNG) [file pone.0263860.s012.png]
