# Supplementary material for: Validation of Friedewald, Martin-Hopkins and Sampson low-density lipoprotein cholesterol equations
Source: PLoS One. 2022 May 13;17(5):e0263860. doi: 10.1371/journal.pone.0263860 (PMC9106156; doi:10.1371/journal.pone.0263860)
Supplement: S1 File — (DOCX) [file pone.0263860.s013.docx]

**Supp.Table 1. Median statistics for the ratio of trygliserides to very low-density lipoprotein cholesterol by the cross table of non-high-density lipoprotein cholesterol and trygliserides calculated from the Turkish population (calculated for Roche direct assay method for 30-cell strata)**

| **TG Levels (mg/dL)** | **Non-HDL-C (mg/dL)** | | | | | |
| --- | --- | --- | --- | --- | --- | --- |
|  | **<100** | **100-129** | **130-159** | **160-189** | **190-219** | **≥220** |
| **<100** | 7.36 | 7.00 | 5.88 | 5.22 | 4.22 | 4.56 |
| **100-149** | 8.19 | 8.06 | 7.67 | 6.95 | 5.95 | 5.16 |
| **150-199** | 8.00 | 8.50 | 8.30 | 7.75 | 7.21 | 5.59 |
| **200-399** | 8.47 | 8.71 | 8.73 | 8.16 | 7.64 | 7.06 |
| **>=400** | 12.16 | 8.54 | 8.90 | 8.26 | 7.54 | 6.92 |

**TG: triglycerides; HDL-C: high-density lipoprotein cholesterol**

**Supp.Table 2. Median statistics for the ratio of trygliserides to very low-density lipoprotein cholesterol by the cross table of non-high-density lipoprotein cholesterol and trygliserides calculated from the Turkish population (calculated for Roche direct assay method for 70-cell strata)**

| **TG Levels (mg/dL)** | **Non-HDL-C (mg/dL)** | | | | | | | | | | | | | |
| --- | --- | --- | --- | --- | --- | --- | --- | --- | --- | --- | --- | --- | --- | --- |
|  | **<100** | **100-109** | **110-119** | **120-129** | **130-139** | **140-149** | **150-159** | **160-169** | **170-179** | **180-189** | **190-199** | **200-209** | **210-219** | **≥220** |
| **<100** | 7.36 | 7.36 | 6.80 | 6.92 | 6.00 | 6.13 | 5.44 | 5.29 | 5.20 | 5.13 | 4.50 | 3.63 | 4.40 | 4.56 |
| **100-149** | 8.19 | 8.13 | 8.17 | 7.89 | 8.00 | 7.81 | 7.24 | 6.95 | 6.95 | 7.15 | 6.40 | 5.84 | 5.30 | 5.16 |
| **150-199** | 8.00 | 8.46 | 8.33 | 8.62 | 8.59 | 8.32 | 8.05 | 7.90 | 7.64 | 7.38 | 7.46 | 7.18 | 6.71 | 5.59 |
| **200-399** | 8.47 | 8.92 | 8.84 | 8.49 | 8.69 | 9.04 | 8.48 | 8.44 | 8.00 | 8.01 | 7.78 | 7.70 | 7.38 | 7.06 |
| **>=400** | 12.16 | 8.70 | 9.04 | 7.53 | 8.71 | 8.90 | 8.94 | 8.42 | 8.34 | 7.93 | 7.70 | 7.31 | 7.66 | 6.92 |

**TG: triglycerides; HDL-C: high-density lipoprotein cholesterol**

**Supp.Table 3. Median statistics for the ratio of trygliserides to very low-density lipoprotein cholesterol by the cross table of non-high-density lipoprotein cholesterol and trygliserides calculated from the Turkish population (calculated for Roche direct assay method for 130-cell strata)**

| **TG Levels (mg/dL)** | **Non-HDL-C (mg/dL)** | | | | | | | | | | | | | | | | | | | | | | | | | |
| --- | --- | --- | --- | --- | --- | --- | --- | --- | --- | --- | --- | --- | --- | --- | --- | --- | --- | --- | --- | --- | --- | --- | --- | --- | --- | --- |
|  | **<100** | **100-104** | **105-109** | **110-114** | **115-119** | **120-124** | **125-129** | **130-134** | **135-139** | **140-144** | **145-149** | **150-154** | **155-159** | **160-164** | **165-169** | **170-174** | **175-179** | **180-184** | **185-189** | **190-194** | **195-199** | **200-204** | **205-209** | **210-214** | **215-219** | **≥220** |
| **<100** | 7.36 | 7.15 | 7.53 | 6.85 | 6.73 | 7.14 | 6.78 | 5.92 | 6.15 | 6.23 | 5.83 | 5.81 | 5.11 | 5.23 | 5.44 | 5.00 | 5.50 | 5.28 | 5.11 | 5.03 | 3.69 | 3.59 | 3.82 | 4.42 | 4.30 | 4.56 |
| **100-149** | 8.19 | 8.50 | 7.86 | 8.41 | 8.00 | 8.12 | 7.68 | 8.00 | 7.88 | 8.18 | 7.40 | 7.31 | 7.15 | 6.76 | 7.35 | 7.19 | 6.60 | 6.94 | 7.33 | 5.96 | 6.60 | 5.93 | 5.55 | 5.26 | 5.33 | 5.16 |
| **150-199** | 8.00 | 8.62 | 8.42 | 8.33 | 8.31 | 8.60 | 8.63 | 9.21 | 8.10 | 8.39 | 8.26 | 8.37 | 7.71 | 8.00 | 7.79 | 7.82 | 7.46 | 7.34 | 7.54 | 7.04 | 7.88 | 7.32 | 7.05 | 6.19 | 7.15 | 5.59 |
| **200-399** | 8.47 | 8.53 | 9.14 | 8.92 | 8.78 | 8.50 | 8.46 | 8.46 | 8.76 | 9.03 | 9.04 | 8.49 | 8.47 | 8.53 | 8.36 | 7.92 | 8.10 | 8.12 | 7.92 | 7.78 | 7.76 | 7.50 | 7.84 | 7.44 | 7.31 | 7.06 |
| **>=400** | 12.16 | 10.26 | 6.95 | 9.10 | 8.79 | 7.53 | 7.53 | 7.68 | 9.57 | 9.05 | 8.52 | 8.90 | 9.18 | 8.75 | 8.26 | 8.15 | 8.50 | 8.28 | 7.55 | 7.81 | 7.60 | 7.22 | 7.44 | 7.74 | 7.47 | 6.92 |

**TG: triglycerides; HDL-C: high-density lipoprotein cholesterol**

**Supp.Table 4. Median statistics for the ratio of trygliserides to very low-density lipoprotein cholesterol by the cross table of non-high-density lipoprotein cholesterol and trygliserides calculated from the Turkish population (calculated for Roche direct assay method for 180-cell strata)**

| **TG Levels (mg/dL)** | **Non-HDL-C (mg/dL)** | | | | | |
| --- | --- | --- | --- | --- | --- | --- |
|  | **<100** | **100-129** | **130-159** | **160-189** | **190-219** | **≥220** |
| **7-49** | 6.00 | 5.22 | 3.50 | 4.43 | 2.11 |  |
| **50-56** | 7.00 | 5.60 | 4.23 | 3.93 | 2.23 | 3.65 |
| **57-61** | 7.13 | 6.67 | 4.83 | 3.69 | 2.35 |  |
| **62-66** | 7.22 | 7.11 | 5.73 | 4.74 | 4.40 | 12.80 |
| **67-71** | 7.67 | 6.41 | 5.38 | 5.00 | 2.63 | 2.09 |
| **72-75** | 7.20 | 6.82 | 5.73 | 5.14 | 4.55 | 2.62 |
| **76-79** | 7.80 | 7.05 | 5.64 | 5.43 | 2.97 | 2.81 |
| **80-83** | 7.36 | 6.88 | 5.86 | 5.63 | 4.21 | 2.65 |
| **84-87** | 7.82 | 7.82 | 6.00 | 5.59 | 5.12 | 8.40 |
| **88-92** | 8.00 | 8.18 | 6.13 | 5.08 | 4.05 | 2.94 |
| **93-96** | 8.55 | 7.15 | 6.52 | 5.53 | 6.33 | 4.89 |
| **97-100** | 7.54 | 7.69 | 6.96 | 6.57 | 5.00 | 6.19 |
| **101-105** | 7.92 | 8.00 | 7.11 | 6.50 | 6.71 | 3.74 |
| **106-110** | 8.27 | 7.33 | 7.64 | 6.47 | 5.40 | 5.00 |
| **111-115** | 8.14 | 7.67 | 7.96 | 6.74 | 5.23 | 4.48 |
| **116-120** | 7.73 | 7.87 | 7.44 | 7.00 | 5.65 | 10.90 |
| **121-126** | 8.40 | 8.13 | 7.35 | 7.09 | 6.15 | 5.59 |
| **127-132** | 8.57 | 8.19 | 7.53 | 7.19 | 6.19 | 5.33 |
| **133-138** | 7.75 | 8.59 | 8.06 | 6.90 | 5.90 | 6.18 |
| **139-146** | 8.26 | 8.75 | 7.94 | 7.28 | 7.42 | 5.32 |
| **147-154** | 7.84 | 8.82 | 8.28 | 7.45 | 6.61 | 5.65 |
| **155-163** | 8.00 | 8.37 | 7.93 | 7.80 | 6.87 | 5.59 |
| **164-173** | 7.86 | 8.38 | 8.05 | 7.59 | 6.58 | 5.43 |
| **174-185** | 7.87 | 8.32 | 8.76 | 8.02 | 7.50 | 5.76 |
| **186-201** | 8.64 | 8.59 | 8.38 | 7.72 | 7.15 | 5.68 |
| **202-220** | 8.15 | 8.76 | 8.83 | 8.20 | 7.59 | 6.77 |
| **221-247** | 8.81 | 8.79 | 8.67 | 8.23 | 7.44 | 7.00 |
| **248-292** | 8.56 | 8.66 | 8.83 | 8.23 | 7.85 | 7.15 |
| **293-399** | 8.76 | 8.69 | 8.68 | 8.05 | 7.75 | 7.15 |
| **>=400** | 12.16 | 8.54 | 8.90 | 8.26 | 7.54 | 6.92 |

**TG: triglycerides; HDL-C: high-density lipoprotein cholesterol**

**Supp.Table 5. Median statistics for the ratio of trygliserides to very low-density lipoprotein cholesterol by the cross table of non-high-density lipoprotein cholesterol and trygliserides calculated from the Turkish population (calculated for Roche direct assay method for 420-cell strata)**

| **TG Levels (mg/dL)** | **Non-HDL-C (mg/dL)** | | | | | | | | | | | | | |
| --- | --- | --- | --- | --- | --- | --- | --- | --- | --- | --- | --- | --- | --- | --- |
|  | **<100** | **100-109** | **110-119** | **120-129** | **130-139** | **140-149** | **150-159** | **160-169** | **170-179** | **180-189** | **190-199** | **200-209** | **210-219** | **≥220** |
| **7-49** | 6.00 | 5.88 | 5.22 | 5.00 | 3.08 | 6.81 | 2.88 | 3.76 | 8.00 | 4.78 |  | 2.11 |  |  |
| **50-56** | 7.00 | 6.17 | 5.67 | 5.20 | 5.60 | 3.24 | 3.76 | 3.64 | 3.54 | 5.15 | 1.17 | 3.29 |  | 3.65 |
| **57-61** | 7.13 | 6.10 | 6.78 | 6.78 | 5.04 | 4.60 | 3.99 | 4.33 | 3.69 | 6.69 | 3.69 | 2.35 | 1.58 |  |
| **62-66** | 7.22 | 7.49 | 7.00 | 7.00 | 5.73 | 6.20 | 5.50 | 4.71 | 3.44 | 4.92 | 17.58 |  | 4.40 | 12.80 |
| **67-71** | 7.67 | 6.58 | 6.09 | 6.09 | 5.15 | 5.27 | 6.36 | 5.27 | 5.16 | 4.49 | 4.01 | 2.27 |  | 2.09 |
| **72-75** | 7.20 | 7.50 | 6.25 | 7.30 | 5.32 | 6.64 | 6.08 | 5.00 | 4.90 | 8.68 | 4.55 | 5.78 |  | 2.62 |
| **76-79** | 7.80 | 8.44 | 6.33 | 6.50 | 6.08 | 5.64 | 4.75 | 5.13 | 5.13 | 9.50 | 3.84 | 2.66 | 2.26 | 2.81 |
| **80-83** | 7.36 | 6.92 | 7.36 | 6.75 | 6.23 | 5.86 | 5.03 | 5.40 | 7.36 | 4.10 | 3.99 | 3.62 | 5.34 | 2.65 |
| **84-87** | 7.82 | 9.50 | 7.25 | 7.21 | 5.73 | 6.14 | 6.00 | 6.30 | 4.79 | 5.25 | 3.63 | 5.97 |  | 8.40 |
| **88-92** | 8.00 | 8.18 | 8.00 | 8.36 | 6.85 | 6.77 | 5.29 | 5.69 | 4.94 | 5.00 | 4.32 | 4.39 | 2.75 | 2.94 |
| **93-96** | 8.55 | 6.79 | 6.71 | 7.79 | 6.56 | 6.86 | 6.23 | 5.35 | 6.79 | 5.21 | 5.53 | 52.32 | 7.31 | 4.89 |
| **97-100** | 7.54 | 8.08 | 8.13 | 7.00 | 7.14 | 7.54 | 5.88 | 6.63 | 5.82 | 7.14 | 5.66 | 4.83 | 10.24 | 6.19 |
| **101-105** | 7.92 | 8.08 | 7.81 | 7.81 | 7.92 | 6.73 | 6.44 | 7.36 | 6.31 | 5.10 | 6.71 | 5.28 | 10.00 | 3.74 |
| **106-110** | 8.27 | 7.64 | 7.07 | 7.49 | 7.82 | 7.71 | 7.17 | 6.84 | 5.81 | 7.27 | 5.38 | 5.17 | 7.64 | 5.00 |
| **111-115** | 8.14 | 7.57 | 8.14 | 7.60 | 8.00 | 7.50 | 7.93 | 6.76 | 6.39 | 8.04 | 6.35 | 4.94 | 4.07 | 4.48 |
| **116-120** | 7.73 | 7.44 | 8.32 | 7.73 | 8.00 | 7.25 | 7.06 | 7.44 | 7.16 | 6.05 | 5.90 | 6.32 | 5.07 | 10.90 |
| **121-126** | 8.40 | 8.10 | 8.07 | 8.20 | 7.24 | 8.27 | 6.83 | 6.94 | 7.35 | 6.31 | 7.29 | 5.86 | 5.84 | 5.59 |
| **127-132** | 8.57 | 8.53 | 8.25 | 7.74 | 8.25 | 7.47 | 7.06 | 7.00 | 7.71 | 7.17 | 5.52 | 6.53 | 7.53 | 5.33 |
| **133-138** | 7.75 | 8.87 | 9.50 | 8.09 | 8.31 | 7.61 | 8.00 | 6.80 | 6.85 | 7.33 | 6.57 | 5.99 | 4.93 | 6.18 |
| **139-146** | 8.26 | 8.81 | 9.43 | 7.94 | 7.94 | 8.59 | 7.61 | 6.93 | 7.28 | 7.72 | 7.63 | 6.35 | 6.04 | 5.32 |
| **147-154** | 7.84 | 8.39 | 8.88 | 9.12 | 8.91 | 8.39 | 7.60 | 7.38 | 7.70 | 7.48 | 8.33 | 6.42 | 4.97 | 5.65 |
| **155-163** | 8.00 | 8.53 | 8.13 | 8.69 | 8.37 | 8.61 | 6.79 | 8.16 | 7.75 | 7.05 | 6.57 | 6.98 | 7.23 | 5.59 |
| **164-173** | 7.86 | 8.19 | 8.19 | 8.92 | 7.90 | 7.82 | 8.30 | 8.20 | 7.26 | 6.84 | 7.06 | 6.50 | 5.72 | 5.43 |
| **174-185** | 7.87 | 8.41 | 7.95 | 8.40 | 8.76 | 9.15 | 8.48 | 8.00 | 7.65 | 8.38 | 7.57 | 7.29 | 7.50 | 5.76 |
| **186-201** | 8.64 | 8.88 | 8.74 | 8.55 | 8.82 | 7.96 | 8.62 | 7.56 | 8.17 | 7.29 | 7.19 | 7.30 | 5.84 | 5.68 |
| **202-220** | 8.15 | 8.60 | 8.76 | 8.76 | 9.09 | 9.81 | 8.15 | 8.67 | 7.89 | 8.04 | 7.87 | 7.72 | 7.39 | 6.77 |
| **221-247** | 8.81 | 8.85 | 9.04 | 8.23 | 8.44 | 8.79 | 8.78 | 8.92 | 7.90 | 7.93 | 7.44 | 7.62 | 7.13 | 7.00 |
| **248-292** | 8.56 | 8.90 | 8.66 | 8.56 | 8.91 | 9.02 | 8.35 | 8.26 | 8.19 | 8.29 | 7.87 | 8.27 | 7.36 | 7.15 |
| **293-399** | 8.76 | 8.97 | 8.82 | 8.43 | 8.61 | 9.03 | 8.45 | 8.23 | 7.98 | 8.01 | 7.95 | 7.65 | 7.50 | 7.15 |
| **>=400** | 12.16 | 8.70 | 9.04 | 7.53 | 8.71 | 8.90 | 8.94 | 8.42 | 8.34 | 7.93 | 7.70 | 7.31 | 7.66 | 6.92 |

**TG: triglycerides; HDL-C: high-density lipoprotein cholesterol**

**Supp.Table 6. Median statistics for the ratio of trygliserides to very low-density lipoprotein cholesterol by the cross table of non-high-density lipoprotein cholesterol and trygliserides calculated from the Turkish population (calculated for Roche direct assay method for 780-cell strata)**

| **TG Levels (mg/dL)** | **Non-HDL-C (mg/dL)** | | | | | | | | | | | | | | | | | | | | | | | | | |
| --- | --- | --- | --- | --- | --- | --- | --- | --- | --- | --- | --- | --- | --- | --- | --- | --- | --- | --- | --- | --- | --- | --- | --- | --- | --- | --- |
|  | **<100** | **100-104** | **105-109** | **110-114** | **115-119** | **120-124** | **125-129** | **130-134** | **135-139** | **140-144** | **145-149** | **150-154** | **155-159** | **160-164** | **165-169** | **170-174** | **175-179** | **180-184** | **185-189** | **190-194** | **195-199** | **200-204** | **205-209** | **210-214** | **215-219** | **≥220** |
| **7-49** | 6.00 | 6.40 | 5.24 | 5.22 | 4.79 | 4.90 | 5.75 | 2.73 | 3.77 | 8.17 | 5.44 | 12.87 | 2.88 |  | 3.76 | 8.00 |  | 4.43 | 4.88 |  |  | 2.41 | 2.09 |  |  |  |
| **50-56** | 7.00 | 7.86 | 5.55 | 5.78 | 5.43 | 6.18 | 4.31 | 5.67 | 5.00 | 3.86 | 2.99 | 3.45 | 5.54 | 2.91 | 3.64 | 3.54 |  |  | 5.15 |  | 1.17 |  | 3.29 |  |  | 3.65 |
| **57-61** | 7.13 | 6.17 | 6.10 | 6.67 | 8.03 | 6.46 | 6.78 | 5.18 | 5.00 | 5.58 | 3.63 | 4.45 | 3.40 | 5.68 | 4.33 | 3.69 |  | 7.31 | 6.54 |  | 3.69 | 2.64 | 2.07 |  | 1.58 |  |
| **62-66** | 7.22 | 6.30 | 8.06 | 5.91 | 8.06 | 6.30 | 7.11 | 5.57 | 5.73 | 7.17 | 5.17 | 6.35 | 4.50 | 3.82 | 4.85 | 2.89 | 7.00 | 4.92 | 2.83 | 31.50 | 3.67 |  |  | 4.40 |  | 12.80 |
| **67-71** | 7.67 | 6.41 | 7.22 | 6.80 | 5.58 | 6.09 | 6.80 | 5.15 | 5.15 | 5.48 | 5.00 | 6.27 | 6.45 | 7.92 | 4.63 | 5.91 | 4.27 | 3.38 | 4.93 | 5.78 | 2.43 | 2.27 |  |  |  | 2.09 |
| **72-75** | 7.20 | 6.55 | 8.11 | 6.82 | 5.54 | 7.35 | 7.20 | 6.64 | 4.93 | 6.73 | 5.62 | 7.30 | 5.00 | 4.90 | 5.14 | 4.93 | 4.87 | 7.20 | 9.25 |  | 4.55 | 9.25 | 2.31 |  |  | 2.62 |
| **76-79** | 7.80 | 9.50 | 7.60 | 7.70 | 6.08 | 6.66 | 6.50 | 5.92 | 6.50 | 5.07 | 5.85 | 4.88 | 3.90 | 5.07 | 5.53 | 4.73 | 5.50 | 9.50 | 7.15 | 5.74 | 2.66 | 2.66 |  | 2.26 |  | 2.81 |
| **80-83** | 7.36 | 5.86 | 9.00 | 8.30 | 6.67 | 6.38 | 6.92 | 6.19 | 6.23 | 5.86 | 5.70 | 4.44 | 5.33 | 5.47 | 5.33 | 9.00 | 7.32 | 3.42 | 4.94 | 4.43 | 3.51 | 3.19 | 4.88 | 4.03 | 16.62 | 2.65 |
| **84-87** | 7.82 | 9.56 | 8.60 | 7.04 | 7.64 | 7.44 | 6.58 | 5.12 | 7.21 | 5.80 | 7.17 | 7.88 | 5.44 | 6.46 | 6.00 | 4.16 | 5.73 | 5.25 | 5.35 | 3.06 | 5.12 | 6.21 | 3.82 |  |  | 8.40 |
| **88-92** | 8.00 | 8.23 | 7.46 | 8.00 | 8.00 | 8.23 | 8.36 | 6.39 | 7.26 | 7.05 | 6.57 | 5.29 | 5.75 | 5.93 | 5.69 | 5.00 | 4.94 | 4.74 | 5.11 | 4.00 | 4.74 |  | 4.39 | 1.51 | 2.75 | 2.94 |
| **93-96** | 8.55 | 6.20 | 7.92 | 6.75 | 6.71 | 9.07 | 6.33 | 5.88 | 7.15 | 6.86 | 7.31 | 6.30 | 6.07 | 5.11 | 5.70 | 6.05 | 6.79 | 5.38 | 5.09 | 5.47 | 6.33 | 52.32 |  | 6.82 | 47.00 | 4.89 |
| **97-100** | 7.54 | 8.08 | 7.97 | 7.00 | 9.00 | 9.80 | 5.85 | 7.58 | 6.67 | 7.58 | 7.07 | 5.56 | 6.00 | 6.25 | 8.17 | 5.82 | 5.59 | 10.89 | 5.03 | 5.98 | 4.23 | 4.76 | 4.90 | 16.17 | 4.30 | 6.19 |
| **101-105** | 7.92 | 8.50 | 8.08 | 7.92 | 7.36 | 8.08 | 7.25 | 9.41 | 7.25 | 7.43 | 5.75 | 6.25 | 6.80 | 6.56 | 9.06 | 7.36 | 6.24 | 5.23 | 4.57 | 7.85 | 6.56 | 4.25 | 19.31 | 7.00 | 13.13 | 3.74 |
| **106-110** | 8.27 | 7.20 | 7.82 | 7.33 | 6.88 | 7.27 | 7.64 | 7.64 | 8.92 | 8.83 | 6.52 | 6.72 | 7.57 | 6.81 | 6.88 | 5.74 | 6.11 | 5.20 | 8.23 | 6.08 | 4.55 | 7.20 | 3.06 | 7.75 | 4.70 | 5.00 |
| **111-115** | 8.14 | 8.85 | 7.29 | 7.67 | 8.65 | 7.27 | 7.93 | 7.93 | 8.14 | 7.19 | 8.85 | 8.18 | 7.40 | 6.76 | 6.92 | 6.59 | 6.00 | 6.61 | 9.06 | 5.29 | 7.13 | 5.65 | 3.73 | 4.20 | 4.07 | 4.48 |
| **116-120** | 7.73 | 7.31 | 7.87 | 8.57 | 7.87 | 7.59 | 7.73 | 7.90 | 8.36 | 6.94 | 7.31 | 7.34 | 6.50 | 7.50 | 7.34 | 8.25 | 5.70 | 5.90 | 6.32 | 5.36 | 6.11 | 5.09 | 6.88 | 5.22 | 4.92 | 10.90 |
| **121-126** | 8.40 | 8.71 | 7.69 | 8.71 | 7.63 | 9.19 | 7.88 | 7.29 | 7.12 | 8.27 | 7.69 | 6.89 | 6.42 | 6.53 | 7.15 | 8.20 | 7.26 | 6.31 | 6.91 | 5.83 | 7.49 | 5.93 | 5.04 | 5.73 | 6.30 | 5.59 |
| **127-132** | 8.57 | 8.90 | 7.11 | 8.09 | 8.39 | 7.94 | 7.47 | 9.04 | 8.06 | 7.28 | 7.97 | 6.84 | 7.11 | 6.95 | 7.08 | 7.00 | 8.53 | 7.76 | 6.19 | 6.05 | 5.29 | 7.22 | 6.35 | 5.84 | 7.53 | 5.33 |
| **133-138** | 7.75 | 8.00 | 9.75 | 9.00 | 9.90 | 8.28 | 7.94 | 8.53 | 8.31 | 7.67 | 7.12 | 8.00 | 8.03 | 6.55 | 7.24 | 7.16 | 6.45 | 8.33 | 6.90 | 7.30 | 6.57 | 6.23 | 5.40 | 4.93 | 5.04 | 6.18 |
| **139-146** | 8.26 | 9.26 | 8.69 | 9.73 | 8.91 | 8.59 | 7.47 | 7.97 | 7.63 | 8.69 | 8.29 | 7.58 | 7.68 | 6.88 | 7.03 | 7.73 | 6.67 | 7.13 | 7.75 | 6.95 | 8.53 | 6.26 | 8.26 | 6.04 | 6.12 | 5.32 |
| **147-154** | 7.84 | 8.06 | 8.47 | 8.82 | 8.94 | 8.56 | 9.25 | 9.50 | 8.28 | 9.19 | 7.75 | 7.55 | 7.65 | 7.00 | 8.22 | 8.64 | 6.04 | 6.82 | 8.50 | 7.33 | 8.90 | 6.42 | 6.03 | 4.63 | 5.50 | 5.65 |
| **155-163** | 8.00 | 8.18 | 8.94 | 7.93 | 8.24 | 8.78 | 8.61 | 8.67 | 8.00 | 7.52 | 9.21 | 6.87 | 6.66 | 7.95 | 8.47 | 8.00 | 7.45 | 7.18 | 6.50 | 6.87 | 6.15 | 7.11 | 6.49 | 7.32 | 6.97 | 5.59 |
| **164-173** | 7.86 | 8.89 | 7.55 | 8.19 | 8.19 | 9.56 | 7.86 | 8.45 | 7.52 | 8.30 | 7.39 | 8.30 | 8.33 | 9.64 | 7.90 | 7.61 | 7.13 | 6.90 | 6.78 | 6.50 | 7.68 | 6.50 | 6.31 | 6.35 | 5.57 | 5.43 |
| **174-185** | 7.87 | 8.41 | 8.36 | 9.60 | 7.38 | 8.41 | 8.36 | 9.34 | 8.36 | 9.42 | 8.88 | 8.95 | 7.63 | 8.04 | 7.83 | 7.48 | 8.04 | 8.30 | 8.62 | 7.04 | 7.71 | 9.26 | 7.12 | 7.27 | 7.91 | 5.76 |
| **186-201** | 8.64 | 8.62 | 8.91 | 8.57 | 8.87 | 8.00 | 8.77 | 9.24 | 8.73 | 8.04 | 7.81 | 8.88 | 8.21 | 7.96 | 7.31 | 8.36 | 7.88 | 7.37 | 6.54 | 6.69 | 7.92 | 8.00 | 7.05 | 5.31 | 7.15 | 5.68 |
| **202-220** | 8.15 | 8.33 | 9.41 | 8.66 | 8.88 | 8.71 | 8.78 | 9.16 | 8.96 | 9.71 | 9.82 | 7.92 | 8.58 | 8.91 | 8.50 | 7.88 | 7.93 | 8.04 | 7.90 | 8.36 | 7.68 | 7.45 | 8.37 | 7.39 | 7.27 | 6.77 |
| **221-247** | 8.81 | 8.64 | 9.08 | 9.04 | 9.15 | 8.23 | 8.26 | 8.28 | 8.59 | 8.85 | 8.78 | 8.78 | 8.70 | 8.89 | 8.92 | 7.66 | 8.00 | 7.79 | 7.97 | 7.23 | 7.63 | 7.50 | 7.68 | 7.13 | 7.26 | 7.00 |
| **248-292** | 8.56 | 8.71 | 9.32 | 8.84 | 8.56 | 8.70 | 8.45 | 8.52 | 9.03 | 9.06 | 9.00 | 8.92 | 8.00 | 8.51 | 7.86 | 7.89 | 8.23 | 8.30 | 7.86 | 8.45 | 7.56 | 8.00 | 8.92 | 7.16 | 7.88 | 7.15 |
| **293-399** | 8.76 | 7.47 | 9.65 | 8.69 | 8.82 | 8.17 | 8.49 | 8.04 | 8.91 | 9.03 | 9.05 | 8.63 | 8.36 | 8.13 | 8.29 | 8.08 | 7.94 | 8.15 | 7.92 | 7.82 | 7.99 | 7.14 | 7.93 | 8.05 | 7.24 | 7.15 |
| **>=400** | 12.16 | 10.26 | 6.95 | 9.10 | 8.79 | 7.53 | 7.53 | 7.68 | 9.57 | 9.05 | 8.52 | 8.90 | 9.18 | 8.75 | 8.26 | 8.15 | 8.50 | 8.28 | 7.55 | 7.81 | 7.60 | 7.22 | 7.44 | 7.74 | 7.47 | 6.92 |

**TG: triglycerides; HDL-C: high-density lipoprotein cholesterol**

**Supp.Table 7. Median statistics for the ratio of trygliserides to very low-density lipoprotein cholesterol by the cross table of non-high-density lipoprotein cholesterol and trygliserides calculated from the Turkish population (calculated for Beckman direct assay method for 30-cell strata)**

| **TG Levels (mg/dL)** | **Non-HDL-C (mg/dL)** | | | | | |
| --- | --- | --- | --- | --- | --- | --- |
|  | **<100** | **100-129** | **130-159** | **160-189** | **190-219** | **≥220** |
| **<100** | 9.71 | 6.93 | 5.27 | 3.84 | 2.96 | 2.48 |
| **100-149** | 13.70 | 9.62 | 7.31 | 5.51 | 4.48 | 3.43 |
| **150-199** | 15.64 | 12.13 | 9.64 | 7.52 | 5.79 | 4.57 |
| **200-399** | 20.78 | 15.94 | 12.06 | 9.78 | 8.11 | 6.24 |
| **>=400** | 23.56 | 17.93 | 15.31 | 12.99 | 11.03 | 8.87 |

**TG: triglycerides; HDL-C: high-density lipoprotein cholesterol**

**Supp.Table 8. Median statistics for the ratio of trygliserides to very low-density lipoprotein cholesterol by the cross table of non-high-density lipoprotein cholesterol and trygliserides calculated from the Turkish population (calculated for Beckman direct assay method for 70-cell strata)**

| **TG Levels (mg/dL)** | **Non-HDL-C (mg/dL)** | | | | | | | | | | | | | |
| --- | --- | --- | --- | --- | --- | --- | --- | --- | --- | --- | --- | --- | --- | --- |
|  | **<100** | **100-109** | **110-119** | **120-129** | **130-139** | **140-149** | **150-159** | **160-169** | **170-179** | **180-189** | **190-199** | **200-209** | **210-219** | **≥220** |
| **<100** | 9.71 | 7.33 | 7.00 | 6.64 | 5.53 | 5.24 | 4.87 | 3.94 | 4.17 | 3.52 | 3.33 | 2.72 | 2.88 | 2.48 |
| **100-149** | 13.70 | 11.00 | 9.71 | 8.60 | 8.00 | 7.27 | 6.67 | 5.76 | 5.64 | 5.00 | 4.83 | 4.45 | 3.94 | 3.43 |
| **150-199** | 15.64 | 13.48 | 12.79 | 10.70 | 10.67 | 9.46 | 9.00 | 8.11 | 7.58 | 6.61 | 6.27 | 5.56 | 5.36 | 4.57 |
| **200-399** | 20.78 | 18.31 | 15.70 | 15.08 | 13.54 | 12.16 | 11.49 | 10.64 | 10.22 | 8.76 | 8.70 | 8.03 | 7.48 | 6.24 |
| **>=400** | 23.56 |  | 20.80 | 17.39 | 17.15 | 14.50 | 14.50 | 14.24 | 12.74 | 12.07 | 12.10 | 10.48 | 10.73 | 8.87 |

**TG: triglycerides; HDL-C: high-density lipoprotein cholesterol**

**Supp.Table 9. Median statistics for the ratio of trygliserides to very low-density lipoprotein cholesterol by the cross table of non-high-density lipoprotein cholesterol and trygliserides calculated from the Turkish population (calculated for Beckman direct assay method for 130-cell strata)**

| **TG Levels (mg/dL)** | **Non-HDL-C (mg/dL)** | | | | | | | | | | | | | | | | | | | | | | | | | |
| --- | --- | --- | --- | --- | --- | --- | --- | --- | --- | --- | --- | --- | --- | --- | --- | --- | --- | --- | --- | --- | --- | --- | --- | --- | --- | --- |
|  | **<100** | **100-104** | **105-109** | **110-114** | **115-119** | **120-124** | **125-129** | **130-134** | **135-139** | **140-144** | **145-149** | **150-154** | **155-159** | **160-164** | **165-169** | **170-174** | **175-179** | **180-184** | **185-189** | **190-194** | **195-199** | **200-204** | **205-209** | **210-214** | **215-219** | **≥220** |
| **<100** | 9.71 | 8.00 | 6.91 | 7.44 | 6.55 | 6.54 | 6.71 | 5.24 | 5.80 | 5.37 | 5.18 | 4.87 | 4.91 | 4.21 | 3.68 | 4.14 | 4.19 | 3.52 | 3.49 | 3.44 | 2.89 | 2.89 | 2.61 | 2.93 | 2.85 | 2.48 |
| **100-149** | 13.70 | 11.33 | 10.83 | 9.95 | 9.62 | 8.60 | 8.60 | 7.94 | 8.19 | 7.10 | 7.46 | 7.00 | 6.57 | 5.98 | 5.67 | 5.72 | 5.45 | 4.96 | 5.18 | 4.96 | 4.81 | 4.48 | 4.39 | 3.86 | 3.97 | 3.43 |
| **150-199** | 15.64 | 13.57 | 13.38 | 12.73 | 13.04 | 10.70 | 10.61 | 11.36 | 10.21 | 9.51 | 9.29 | 9.29 | 8.77 | 9.00 | 7.59 | 7.86 | 7.09 | 6.57 | 6.64 | 6.76 | 5.30 | 6.17 | 5.12 | 5.45 | 5.32 | 4.57 |
| **200-399** | 20.78 | 19.71 | 18.14 | 16.83 | 15.44 | 15.95 | 13.93 | 13.17 | 14.06 | 12.53 | 11.27 | 11.59 | 11.18 | 11.22 | 10.11 | 9.87 | 10.39 | 9.09 | 8.43 | 9.04 | 8.41 | 8.13 | 7.81 | 7.17 | 7.79 | 6.24 |
| **>=400** | 23.56 |  |  | 28.24 | 18.46 | 17.39 | 19.40 | 18.17 | 15.60 | 17.44 | 13.91 | 14.01 | 15.15 | 14.57 | 13.92 | 12.88 | 12.58 | 13.12 | 11.20 | 12.51 | 12.09 | 11.36 | 8.97 | 10.58 | 10.73 | 8.87 |

**TG: triglycerides; HDL-C: high-density lipoprotein cholesterol**

**Supp.Table 10. Median statistics for the ratio of trygliserides to very low-density lipoprotein cholesterol by the cross table of non-high-density lipoprotein cholesterol and trygliserides calculated from the Turkish population (calculated for Beckman direct assay method for 180-cell strata)**

| **TG Levels (mg/dL)** | **Non-HDL-C (mg/dL)** | | | | | |
| --- | --- | --- | --- | --- | --- | --- |
|  | **<100** | **100-129** | **130-159** | **160-189** | **190-219** | **≥220** |
| **7-49** | 6.82 | 4.04 | 3.91 | 2.01 | 2.07 | 1.02 |
| **50-56** | 8.17 | 5.00 | 3.50 | 2.24 | 2.83 | 1.98 |
| **57-61** | 9.50 | 6.10 | 4.46 | 3.63 | 2.68 | 3.05 |
| **62-66** | 9.88 | 6.40 | 4.77 | 2.71 | 2.54 | 1.99 |
| **67-71** | 9.57 | 7.05 | 4.63 | 3.55 | 2.65 | 1.43 |
| **72-75** | 9.25 | 7.40 | 4.93 | 4.00 | 2.64 | 2.11 |
| **76-79** | 9.75 | 7.70 | 5.64 | 3.71 | 2.75 | 2.60 |
| **80-83** | 13.33 | 7.77 | 5.19 | 4.37 | 2.85 | 2.35 |
| **84-87** | 10.88 | 7.17 | 5.67 | 3.58 | 3.19 | 2.97 |
| **88-92** | 12.04 | 7.42 | 5.93 | 4.74 | 3.37 | 2.49 |
| **93-96** | 10.56 | 8.00 | 5.88 | 4.36 | 3.72 | 3.47 |
| **97-100** | 12.38 | 8.82 | 6.53 | 4.81 | 3.59 | 2.56 |
| **101-105** | 11.44 | 8.58 | 6.44 | 5.00 | 4.21 | 2.81 |
| **106-110** | 12.06 | 9.08 | 6.78 | 5.24 | 3.79 | 3.03 |
| **111-115** | 12.33 | 9.25 | 7.13 | 4.87 | 4.11 | 3.02 |
| **116-120** | 12.00 | 9.19 | 6.82 | 5.27 | 4.46 | 3.08 |
| **121-126** | 15.75 | 10.25 | 7.69 | 5.70 | 4.54 | 3.97 |
| **127-132** | 15.94 | 9.77 | 7.94 | 5.52 | 4.40 | 3.49 |
| **133-138** | 14.78 | 10.42 | 7.44 | 6.11 | 5.06 | 3.62 |
| **139-146** | 16.06 | 10.21 | 8.29 | 5.88 | 5.15 | 3.75 |
| **147-154** | 15.92 | 11.38 | 8.44 | 6.67 | 4.78 | 3.76 |
| **155-163** | 17.44 | 11.43 | 9.53 | 6.87 | 5.61 | 4.45 |
| **164-173** | 15.41 | 12.69 | 9.88 | 7.26 | 5.39 | 5.34 |
| **174-185** | 16.27 | 12.71 | 9.89 | 7.36 | 6.07 | 4.14 |
| **186-201** | 18.09 | 12.87 | 10.34 | 8.78 | 6.46 | 5.03 |
| **202-220** | 18.07 | 14.71 | 11.16 | 8.46 | 6.66 | 5.81 |
| **221-247** | 21.27 | 15.77 | 11.60 | 9.04 | 7.09 | 5.66 |
| **248-292** | 20.77 | 15.78 | 12.13 | 10.00 | 8.29 | 6.16 |
| **293-399** | 24.86 | 19.15 | 14.77 | 11.44 | 9.45 | 6.83 |
| **>=400** | 23.56 | 17.93 | 15.31 | 12.99 | 11.03 | 8.87 |

**TG: triglycerides; HDL-C: high-density lipoprotein cholesterol**

**Supp.Table 11. Median statistics for the ratio of trygliserides to very low-density lipoprotein cholesterol by the cross table of non-high-density lipoprotein cholesterol and trygliserides calculated from the Turkish population (calculated for Beckman direct assay method for 420-cell strata)**

| **TG Levels (mg/dL)** | **Non-HDL-C (mg/dL)** | | | | | | | | | | | | | |
| --- | --- | --- | --- | --- | --- | --- | --- | --- | --- | --- | --- | --- | --- | --- |
|  | **<100** | **100-109** | **110-119** | **120-129** | **130-139** | **140-149** | **150-159** | **160-169** | **170-179** | **180-189** | **190-199** | **200-209** | **210-219** | **≥220** |
| **7-49** | 6.82 | 5.04 | 4.00 | 3.23 | 4.08 | 4.00 | 2.80 | 1.82 | 3.66 | 2.27 | 2.04 | 4.00 | 1.43 | 1.02 |
| **50-56** | 8.17 | 5.60 | 4.28 | 4.38 | 4.08 | 4.20 | 2.30 | 2.60 | 2.17 | 1.96 | 2.74 | 1.90 | 4.77 | 1.98 |
| **57-61** | 9.50 | 7.31 | 6.10 | 5.04 | 4.69 | 4.14 | 4.49 | 4.60 | 3.96 | 2.01 | 3.75 | 1.36 | 2.19 | 3.05 |
| **62-66** | 9.88 | 7.54 | 6.40 | 5.29 | 4.74 | 5.17 | 4.33 | 2.56 | 3.15 | 2.54 | 2.30 | 2.83 | 2.36 | 1.99 |
| **67-71** | 9.57 | 7.44 | 7.05 | 6.45 | 5.58 | 4.52 | 3.94 | 3.54 | 3.70 | 2.69 | 2.82 | 2.59 | 2.80 | 1.43 |
| **72-75** | 9.25 | 7.25 | 7.20 | 8.06 | 4.97 | 4.87 | 5.00 | 4.21 | 4.29 | 2.40 | 2.61 | 2.01 | 4.55 | 2.11 |
| **76-79** | 9.75 | 8.44 | 8.61 | 6.54 | 6.08 | 5.43 | 5.07 | 4.59 | 3.35 | 2.42 | 2.96 | 2.00 | 2.62 | 2.60 |
| **80-83** | 13.33 | 8.15 | 7.55 | 7.45 | 5.93 | 5.13 | 4.69 | 4.21 | 4.63 | 4.43 | 3.24 | 2.60 | 2.13 | 2.35 |
| **84-87** | 10.88 | 8.60 | 7.25 | 7.08 | 5.55 | 5.80 | 5.60 | 3.48 | 3.83 | 3.76 | 3.35 | 3.19 | 2.58 | 2.97 |
| **88-92** | 12.04 | 7.58 | 7.50 | 7.33 | 6.77 | 5.93 | 5.11 | 4.60 | 5.06 | 4.29 | 4.34 | 2.65 | 3.41 | 2.49 |
| **93-96** | 10.56 | 8.00 | 9.30 | 7.38 | 5.94 | 6.07 | 5.62 | 4.52 | 4.43 | 4.04 | 4.13 | 3.20 | 2.91 | 3.47 |
| **97-100** | 12.38 | 9.70 | 9.70 | 7.58 | 6.53 | 7.07 | 6.25 | 5.11 | 4.41 | 4.88 | 4.00 | 3.38 | 3.21 | 2.56 |
| **101-105** | 11.44 | 9.50 | 7.36 | 8.54 | 6.22 | 6.80 | 6.00 | 4.75 | 4.68 | 5.32 | 4.75 | 3.66 | 3.26 | 2.81 |
| **106-110** | 12.06 | 10.70 | 9.68 | 8.15 | 7.86 | 6.69 | 5.57 | 5.54 | 5.79 | 4.15 | 4.24 | 3.31 | 3.48 | 3.03 |
| **111-115** | 12.33 | 10.36 | 10.36 | 7.47 | 8.69 | 5.43 | 7.16 | 5.26 | 4.91 | 4.42 | 4.56 | 3.99 | 3.70 | 3.02 |
| **116-120** | 12.00 | 12.89 | 8.43 | 8.50 | 7.93 | 5.95 | 7.03 | 5.76 | 5.20 | 5.09 | 4.46 | 5.32 | 3.96 | 3.08 |
| **121-126** | 15.75 | 12.55 | 9.54 | 8.71 | 7.75 | 8.30 | 7.12 | 6.68 | 5.68 | 4.32 | 4.79 | 4.73 | 4.13 | 3.97 |
| **127-132** | 15.94 | 9.81 | 10.92 | 9.18 | 8.13 | 8.63 | 6.71 | 6.12 | 5.29 | 5.36 | 4.55 | 4.18 | 4.27 | 3.49 |
| **133-138** | 14.78 | 11.25 | 10.38 | 9.68 | 10.01 | 7.64 | 6.48 | 5.81 | 7.21 | 5.89 | 5.78 | 5.17 | 3.97 | 3.62 |
| **139-146** | 16.06 | 13.00 | 11.38 | 9.43 | 10.07 | 8.69 | 7.47 | 5.94 | 6.36 | 5.19 | 5.92 | 4.80 | 4.42 | 3.75 |
| **147-154** | 15.92 | 11.81 | 12.33 | 10.23 | 10.03 | 8.44 | 7.37 | 7.39 | 6.45 | 6.27 | 4.84 | 4.74 | 4.63 | 3.76 |
| **155-163** | 17.44 | 12.54 | 12.00 | 9.47 | 10.53 | 9.56 | 7.75 | 8.32 | 6.60 | 5.78 | 6.12 | 5.19 | 5.32 | 4.45 |
| **164-173** | 15.41 | 13.00 | 14.95 | 11.93 | 11.13 | 9.08 | 10.12 | 7.00 | 8.10 | 6.92 | 5.83 | 5.02 | 5.60 | 5.34 |
| **174-185** | 16.27 | 14.12 | 14.83 | 10.38 | 11.93 | 9.89 | 8.27 | 7.74 | 7.29 | 6.46 | 6.83 | 5.75 | 4.92 | 4.14 |
| **186-201** | 18.09 | 15.42 | 13.00 | 11.81 | 9.60 | 10.58 | 10.26 | 9.80 | 8.75 | 7.73 | 6.71 | 6.16 | 6.75 | 5.03 |
| **202-220** | 18.07 | 16.12 | 14.60 | 14.17 | 11.53 | 11.89 | 10.84 | 8.79 | 8.81 | 7.85 | 6.59 | 7.67 | 5.85 | 5.81 |
| **221-247** | 21.27 | 19.01 | 13.76 | 13.50 | 13.00 | 11.43 | 10.90 | 9.38 | 8.96 | 8.14 | 8.07 | 6.64 | 6.72 | 5.66 |
| **248-292** | 20.77 | 18.14 | 15.78 | 15.24 | 13.05 | 11.75 | 11.78 | 10.78 | 10.15 | 9.28 | 9.43 | 7.92 | 7.45 | 6.16 |
| **293-399** | 24.86 | 19.59 | 20.07 | 17.43 | 16.54 | 15.06 | 13.82 | 12.05 | 11.86 | 9.77 | 9.91 | 9.47 | 8.68 | 6.83 |
| **>=400** | 23.56 |  | 20.80 | 17.39 | 17.15 | 14.50 | 14.50 | 14.24 | 12.74 | 12.07 | 12.10 | 10.48 | 10.73 | 8.87 |

**TG: triglycerides; HDL-C: high-density lipoprotein cholesterol**

**Supp.Table 12. Median statistics for the ratio of trygliserides to very low-density lipoprotein cholesterol by the cross table of non-high-density lipoprotein cholesterol and trygliserides calculated from the Turkish population (calculated for Beckman direct assay method for 780-cell strata)**

| **TG Levels (mg/dL)** | **Non-HDL-C (mg/dL)** | | | | | | | | | | | | | | | | | | | | | | | | | |
| --- | --- | --- | --- | --- | --- | --- | --- | --- | --- | --- | --- | --- | --- | --- | --- | --- | --- | --- | --- | --- | --- | --- | --- | --- | --- | --- |
|  | **<100** | **100-104** | **105-109** | **110-114** | **115-119** | **120-124** | **125-129** | **130-134** | **135-139** | **140-144** | **145-149** | **150-154** | **155-159** | **160-164** | **165-169** | **170-174** | **175-179** | **180-184** | **185-189** | **190-194** | **195-199** | **200-204** | **205-209** | **210-214** | **215-219** | **≥220** |
| **7-49** | 6.82 | 5.57 | 3.92 | 3.46 | 4.33 | 2.94 | 4.82 | 3.21 | 4.45 | 4.00 | 4.05 | 2.96 | 2.70 | 1.92 | 1.68 | 2.09 | 5.22 | 3.15 | 1.39 | 2.71 | 1.36 |  | 4.00 |  | 1.43 | 1.02 |
| **50-56** | 8.17 | 5.59 | 5.60 | 4.66 | 4.25 | 3.68 | 5.08 | 2.80 | 4.33 | 4.29 | 4.20 | 2.30 | 2.30 | 2.67 | 2.52 | 2.43 | 2.08 | 2.24 | 1.57 | 2.20 | 4.15 | .96 | 2.83 | 5.00 | 4.55 | 1.98 |
| **57-61** | 9.50 | 8.43 | 6.56 | 6.95 | 5.55 | 5.18 | 4.69 | 4.54 | 5.27 | 4.83 | 3.43 | 3.37 | 5.00 | 4.29 | 4.92 | 3.00 | 5.45 | 2.07 | 1.70 | 7.18 | 3.75 | 1.29 | 2.03 | 5.38 | 2.14 | 3.05 |
| **62-66** | 9.88 | 7.32 | 7.67 | 6.35 | 7.00 | 5.13 | 5.77 | 5.46 | 4.23 | 5.45 | 5.17 | 4.40 | 4.23 | 2.74 | 2.34 | 3.10 | 3.82 | 2.52 | 2.54 | 2.95 | 1.91 | 3.18 | 2.83 | 12.01 | 1.58 | 1.99 |
| **67-71** | 9.57 | 9.79 | 6.90 | 7.10 | 6.90 | 6.27 | 6.63 | 4.86 | 6.36 | 5.15 | 4.38 | 3.83 | 4.13 | 3.54 | 3.64 | 3.20 | 4.59 | 2.30 | 3.45 | 3.33 | 2.16 | 3.73 | 2.58 | 4.12 | 2.80 | 1.43 |
| **72-75** | 9.25 | 8.28 | 6.17 | 7.45 | 6.08 | 7.32 | 8.11 | 4.63 | 6.08 | 5.69 | 4.20 | 5.31 | 4.93 | 5.52 | 3.39 | 5.77 | 3.65 | 2.31 | 2.48 | 2.61 | 2.58 | 1.99 | 2.49 | 40.35 | 3.23 | 2.11 |
| **76-79** | 9.75 | 8.56 | 7.70 | 9.08 | 7.65 | 6.75 | 6.42 | 7.70 | 4.94 | 6.91 | 4.94 | 5.88 | 3.95 | 5.28 | 3.95 | 3.29 | 3.62 | 2.15 | 2.63 | 2.96 | 2.60 | 3.57 | 2.00 | 2.23 | 4.22 | 2.60 |
| **80-83** | 13.33 | 6.92 | 9.22 | 8.15 | 7.27 | 7.55 | 6.83 | 5.53 | 6.08 | 5.03 | 5.19 | 4.32 | 5.71 | 4.34 | 3.90 | 4.71 | 4.56 | 4.43 | 3.82 | 3.04 | 3.68 | 3.46 | 2.19 | 2.93 | 1.91 | 2.35 |
| **84-87** | 10.88 | 9.33 | 6.85 | 8.45 | 6.62 | 7.08 | 7.00 | 5.44 | 5.67 | 7.25 | 4.60 | 5.73 | 5.00 | 3.50 | 3.39 | 3.31 | 5.12 | 3.60 | 3.89 | 3.70 | 2.81 | 3.19 | 3.57 | 3.03 | 2.58 | 2.97 |
| **88-92** | 12.04 | 7.46 | 8.00 | 8.27 | 6.92 | 7.00 | 7.38 | 6.03 | 7.33 | 6.00 | 5.90 | 4.75 | 5.18 | 4.64 | 4.24 | 5.20 | 3.52 | 4.19 | 4.38 | 4.94 | 2.97 | 2.65 | 4.34 | 3.00 | 3.71 | 2.49 |
| **93-96** | 10.56 | 10.39 | 6.79 | 9.97 | 7.92 | 8.62 | 7.15 | 5.53 | 6.71 | 5.31 | 6.52 | 5.65 | 5.59 | 4.40 | 4.52 | 4.04 | 4.50 | 3.69 | 6.20 | 4.55 | 2.58 | 3.72 | 2.71 | 2.75 | 5.81 | 3.47 |
| **97-100** | 12.38 | 10.89 | 8.91 | 9.90 | 8.91 | 7.50 | 8.29 | 6.67 | 6.30 | 6.60 | 7.46 | 7.06 | 5.76 | 5.84 | 4.67 | 4.50 | 3.73 | 5.16 | 4.30 | 4.08 | 3.92 | 3.96 | 2.51 | 3.13 | 3.30 | 2.56 |
| **101-105** | 11.44 | 9.10 | 9.55 | 7.77 | 7.15 | 9.38 | 7.75 | 7.92 | 5.25 | 8.08 | 5.45 | 6.41 | 5.61 | 4.73 | 5.72 | 6.77 | 4.33 | 5.34 | 5.26 | 4.56 | 5.42 | 3.50 | 3.81 | 3.26 | 3.43 | 2.81 |
| **106-110** | 12.06 | 7.75 | 11.00 | 9.77 | 9.68 | 6.11 | 8.92 | 7.86 | 7.94 | 6.29 | 7.17 | 5.35 | 6.47 | 6.81 | 5.05 | 6.06 | 4.98 | 4.82 | 3.52 | 3.89 | 5.76 | 6.33 | 3.07 | 2.52 | 3.72 | 3.03 |
| **111-115** | 12.33 | 11.50 | 9.84 | 10.36 | 10.88 | 6.76 | 7.50 | 8.54 | 9.33 | 5.43 | 6.01 | 7.50 | 6.65 | 4.63 | 5.89 | 4.87 | 5.23 | 4.35 | 4.64 | 4.51 | 4.56 | 3.39 | 5.43 | 3.36 | 4.65 | 3.02 |
| **116-120** | 12.00 | 13.92 | 10.82 | 8.43 | 8.25 | 8.36 | 8.57 | 7.47 | 8.64 | 6.21 | 5.58 | 7.93 | 6.06 | 6.52 | 5.30 | 4.45 | 6.03 | 5.52 | 4.46 | 4.69 | 3.87 | 6.01 | 3.50 | 3.52 | 4.46 | 3.08 |
| **121-126** | 15.75 | 15.25 | 11.27 | 8.71 | 10.29 | 7.55 | 9.31 | 7.35 | 8.79 | 7.75 | 8.40 | 7.12 | 7.21 | 6.83 | 6.30 | 5.73 | 5.04 | 3.82 | 5.67 | 3.88 | 4.85 | 4.31 | 4.92 | 4.03 | 5.25 | 3.97 |
| **127-132** | 15.94 | 9.96 | 9.56 | 11.91 | 9.83 | 9.39 | 7.79 | 7.94 | 8.19 | 7.06 | 9.07 | 7.28 | 6.60 | 5.65 | 6.14 | 5.28 | 5.41 | 5.98 | 5.10 | 4.57 | 4.54 | 4.57 | 3.18 | 6.12 | 3.63 | 3.49 |
| **133-138** | 14.78 | 11.25 | 11.29 | 10.48 | 10.38 | 10.31 | 9.00 | 11.33 | 9.07 | 6.65 | 8.00 | 6.27 | 6.52 | 5.96 | 5.63 | 6.37 | 7.35 | 6.38 | 5.83 | 5.91 | 4.68 | 5.29 | 4.92 | 3.97 | 5.08 | 3.62 |
| **139-146** | 16.06 | 13.18 | 10.92 | 11.92 | 10.21 | 9.60 | 9.27 | 8.11 | 10.85 | 10.14 | 8.09 | 7.05 | 8.41 | 6.58 | 5.38 | 6.36 | 6.35 | 5.30 | 5.19 | 6.62 | 5.46 | 4.21 | 5.35 | 4.96 | 3.92 | 3.75 |
| **147-154** | 15.92 | 12.58 | 11.46 | 11.20 | 12.50 | 8.75 | 10.38 | 9.85 | 10.03 | 9.16 | 8.28 | 7.24 | 7.50 | 7.29 | 7.55 | 6.91 | 5.86 | 5.73 | 7.45 | 5.47 | 4.58 | 5.09 | 4.08 | 4.52 | 4.74 | 3.76 |
| **155-163** | 17.44 | 16.26 | 10.40 | 13.08 | 10.40 | 9.41 | 9.91 | 11.36 | 10.20 | 9.56 | 9.46 | 8.61 | 7.23 | 10.00 | 7.02 | 7.85 | 5.79 | 5.89 | 5.71 | 6.12 | 6.12 | 5.91 | 4.49 | 6.25 | 4.88 | 4.45 |
| **164-173** | 15.41 | 12.85 | 13.23 | 18.33 | 12.29 | 11.86 | 11.93 | 11.33 | 10.12 | 8.84 | 9.17 | 9.94 | 10.18 | 6.92 | 7.13 | 7.45 | 8.84 | 7.57 | 6.24 | 7.00 | 4.45 | 4.96 | 5.02 | 5.21 | 5.75 | 5.34 |
| **174-185** | 16.27 | 13.21 | 14.15 | 12.39 | 17.50 | 10.26 | 11.53 | 12.20 | 11.60 | 9.94 | 9.83 | 8.20 | 8.29 | 8.27 | 7.50 | 7.96 | 6.29 | 6.20 | 6.46 | 7.42 | 6.21 | 6.23 | 4.56 | 5.55 | 4.31 | 4.14 |
| **186-201** | 18.09 | 13.71 | 19.50 | 12.06 | 14.07 | 11.81 | 11.57 | 10.00 | 9.50 | 11.30 | 10.44 | 10.21 | 10.34 | 10.11 | 9.75 | 8.59 | 9.00 | 7.65 | 7.84 | 6.82 | 5.96 | 6.32 | 5.88 | 5.25 | 7.48 | 5.03 |
| **202-220** | 18.07 | 15.26 | 17.32 | 14.60 | 14.62 | 15.64 | 13.53 | 12.06 | 11.43 | 12.35 | 10.35 | 11.37 | 9.59 | 11.11 | 8.04 | 8.50 | 9.27 | 7.98 | 7.57 | 6.97 | 6.17 | 7.56 | 8.08 | 5.75 | 5.85 | 5.81 |
| **221-247** | 21.27 | 23.10 | 16.79 | 13.21 | 13.76 | 15.93 | 12.63 | 12.89 | 14.00 | 12.39 | 11.07 | 11.79 | 8.50 | 9.84 | 9.07 | 8.81 | 9.08 | 8.61 | 7.80 | 7.66 | 8.20 | 7.34 | 6.16 | 6.02 | 7.57 | 5.66 |
| **248-292** | 20.77 | 16.37 | 18.14 | 19.54 | 15.26 | 17.06 | 14.12 | 12.73 | 13.05 | 11.38 | 12.03 | 11.81 | 11.75 | 10.75 | 10.78 | 9.67 | 10.42 | 9.14 | 9.62 | 9.59 | 9.21 | 8.62 | 7.35 | 7.15 | 7.45 | 6.16 |
| **293-399** | 24.86 | 23.82 | 18.31 | 20.08 | 20.07 | 15.95 | 19.24 | 17.35 | 16.43 | 15.35 | 14.14 | 12.62 | 13.85 | 13.08 | 11.38 | 11.79 | 11.94 | 10.46 | 9.26 | 10.50 | 9.58 | 9.25 | 9.74 | 8.51 | 8.89 | 6.83 |
| **>=400** | 23.56 |  |  | 28.24 | 18.46 | 17.39 | 19.40 | 18.17 | 15.60 | 17.44 | 13.91 | 14.01 | 15.15 | 14.57 | 13.92 | 12.88 | 12.58 | 13.12 | 11.20 | 12.51 | 12.09 | 11.36 | 8.97 | 10.58 | 10.73 | 8.87 |

**TG: triglycerides; HDL-C: high-density lipoprotein cholesterol**

**Supp.Table 13. Median statistics for the ratio of trygliserides to very low-density lipoprotein cholesterol by the cross table of non-high-density lipoprotein cholesterol and trygliserides calculated from the Turkish population (calculated for Siemens direct assay method for 30-cell strata)**

| **TG Levels (mg/dL)** | **Non-HDL-C (mg/dL)** | | | | | |
| --- | --- | --- | --- | --- | --- | --- |
|  | **<100** | **100-129** | **130-159** | **160-189** | **190-219** | **≥220** |
| **<100** | 3.52 | 3.12 | 2.90 | 2.65 | 1.91 | 1.53 |
| **100-149** | 5.14 | 4.40 | 4.15 | 3.68 | 3.34 | 2.76 |
| **150-199** | 6.00 | 5.45 | 4.95 | 4.42 | 4.03 | 3.36 |
| **200-399** | 6.47 | 6.51 | 5.92 | 5.37 | 5.02 | 4.51 |
| **>=400** | 8.83 | 7.45 | 7.03 | 6.51 | 6.16 | 4.59 |

**TG: triglycerides; HDL-C: high-density lipoprotein cholesterol**

**Supp.Table 14. Median statistics for the ratio of trygliserides to very low-density lipoprotein cholesterol by the cross table of non-high-density lipoprotein cholesterol and trygliserides calculated from the Turkish population (calculated for Siemens direct assay method for 70-cell strata)**

| **TG Levels (mg/dL)** | **Non-HDL-C (mg/dL)** | | | | | | | | | | | | | |
| --- | --- | --- | --- | --- | --- | --- | --- | --- | --- | --- | --- | --- | --- | --- |
|  | **<100** | **100-109** | **110-119** | **120-129** | **130-139** | **140-149** | **150-159** | **160-169** | **170-179** | **180-189** | **190-199** | **200-209** | **210-219** | **≥220** |
| **<100** | 3.52 | 3.27 | 3.13 | 3.03 | 3.00 | 2.84 | 2.62 | 2.93 | 2.63 | 2.34 | 2.31 | 1.70 | 1.78 | 1.53 |
| **100-149** | 5.14 | 4.53 | 4.44 | 4.28 | 4.17 | 4.12 | 4.21 | 3.59 | 3.76 | 3.54 | 3.38 | 3.34 | 3.10 | 2.76 |
| **150-199** | 6.00 | 5.76 | 5.57 | 5.13 | 5.26 | 4.78 | 4.81 | 4.62 | 4.25 | 4.33 | 4.41 | 3.72 | 3.91 | 3.36 |
| **200-399** | 6.47 | 6.80 | 6.39 | 6.41 | 5.99 | 5.94 | 5.86 | 5.56 | 5.39 | 5.13 | 5.20 | 5.03 | 4.73 | 4.51 |
| **>=400** | 8.83 | 8.09 | 7.53 | 7.20 | 7.31 | 7.20 | 6.50 | 6.94 | 7.49 | 5.59 | 6.28 | 6.42 | 5.77 | 4.59 |

**TG: triglycerides; HDL-C: high-density lipoprotein cholesterol**

**Supp.Table 15. Median statistics for the ratio of trygliserides to very low-density lipoprotein cholesterol by the cross table of non-high-density lipoprotein cholesterol and trygliserides calculated from the Turkish population (calculated for Siemens direct assay method for 130-cell strata)**

| **TG Levels (mg/dL)** | **Non-HDL-C (mg/dL)** | | | | | | | | | | | | | | | | | | | | | | | | | |
| --- | --- | --- | --- | --- | --- | --- | --- | --- | --- | --- | --- | --- | --- | --- | --- | --- | --- | --- | --- | --- | --- | --- | --- | --- | --- | --- |
|  | **<100** | **100-104** | **105-109** | **110-114** | **115-119** | **120-124** | **125-129** | **130-134** | **135-139** | **140-144** | **145-149** | **150-154** | **155-159** | **160-164** | **165-169** | **170-174** | **175-179** | **180-184** | **185-189** | **190-194** | **195-199** | **200-204** | **205-209** | **210-214** | **215-219** | **≥220** |
| **<100** | 3.52 | 3.18 | 3.33 | 3.08 | 3.20 | 3.12 | 2.96 | 2.99 | 3.04 | 2.69 | 2.93 | 2.49 | 2.71 | 2.89 | 2.94 | 3.03 | 2.50 | 2.43 | 2.23 | 2.31 | 2.39 | 1.82 | 1.67 | 2.02 | .99 | 1.53 |
| **100-149** | 5.14 | 4.40 | 4.70 | 4.46 | 4.42 | 4.33 | 4.23 | 4.19 | 4.08 | 4.30 | 3.93 | 4.25 | 4.01 | 3.57 | 3.81 | 3.77 | 3.73 | 3.37 | 3.74 | 3.35 | 3.40 | 3.37 | 3.24 | 3.10 | 3.11 | 2.76 |
| **150-199** | 6.00 | 5.74 | 5.77 | 5.37 | 5.73 | 5.14 | 5.13 | 5.54 | 5.03 | 4.66 | 4.93 | 4.79 | 4.86 | 4.72 | 4.60 | 4.47 | 3.86 | 3.83 | 4.69 | 4.58 | 4.08 | 3.83 | 3.67 | 3.56 | 5.21 | 3.36 |
| **200-399** | 6.47 | 6.81 | 6.80 | 6.58 | 6.26 | 6.34 | 6.50 | 5.85 | 6.23 | 6.31 | 5.71 | 5.86 | 5.89 | 5.54 | 5.56 | 5.39 | 5.44 | 5.23 | 5.01 | 5.11 | 5.37 | 5.24 | 4.82 | 4.81 | 4.73 | 4.51 |
| **>=400** | 8.83 | 7.52 | 9.43 | 6.37 | 8.95 | 7.50 | 7.01 | 7.25 | 7.37 | 7.89 | 7.03 | 7.14 | 6.31 | 5.99 | 7.37 | 8.17 | 7.06 | 5.99 | 5.53 | 6.55 | 6.25 | 6.29 | 6.58 | 5.94 | 5.41 | 4.59 |

**TG: triglycerides; HDL-C: high-density lipoprotein cholesterol**

**Supp.Table 16. Median statistics for the ratio of trygliserides to very low-density lipoprotein cholesterol by the cross table of non-high-density lipoprotein cholesterol and trygliserides calculated from the Turkish population (calculated for Siemens direct assay method for 180-cell strata)**

| **TG Levels (mg/dL)** | **Non-HDL-C (mg/dL)** | | | | | |
| --- | --- | --- | --- | --- | --- | --- |
|  | **<100** | **100-129** | **130-159** | **160-189** | **190-219** | **≥220** |
| **7-49** | 2.34 | 2.10 | 1.50 | 1.21 | .62 |  |
| **50-56** | 3.11 | 2.47 | 1.92 | 2.15 | .98 | .39 |
| **57-61** | 3.20 | 2.73 | 2.32 | 2.63 | 1.21 | .99 |
| **62-66** | 3.37 | 2.52 | 2.17 | 2.46 | 1.41 |  |
| **67-71** | 3.68 | 2.84 | 2.53 | 2.18 | 2.92 |  |
| **72-75** | 3.75 | 3.13 | 2.71 | 2.03 | 1.77 |  |
| **76-79** | 3.66 | 3.14 | 3.16 | 2.71 | 2.35 | .80 |
| **80-83** | 3.91 | 3.47 | 2.89 | 3.20 | 1.74 | 2.37 |
| **84-87** | 4.25 | 3.41 | 3.26 | 2.29 | 1.49 | 1.76 |
| **88-92** | 4.09 | 3.83 | 3.54 | 2.73 | 1.97 | 1.75 |
| **93-96** | 4.43 | 3.96 | 3.43 | 3.29 | 3.77 | 1.08 |
| **97-100** | 4.25 | 3.90 | 3.05 | 3.05 | 2.19 | 1.95 |
| **101-105** | 4.81 | 4.13 | 3.68 | 2.94 | 3.76 | 2.25 |
| **106-110** | 4.91 | 4.09 | 3.53 | 2.69 | 2.69 | 3.72 |
| **111-115** | 5.14 | 4.63 | 3.99 | 3.35 | 3.29 | 3.06 |
| **116-120** | 5.29 | 4.33 | 4.20 | 3.55 | 3.53 | 3.24 |
| **121-126** | 5.30 | 4.31 | 4.25 | 3.73 | 2.80 | 2.41 |
| **127-132** | 5.30 | 4.45 | 4.21 | 3.78 | 3.12 | 3.11 |
| **133-138** | 5.04 | 4.74 | 4.15 | 3.79 | 3.45 | 2.84 |
| **139-146** | 5.33 | 4.71 | 4.77 | 4.25 | 3.47 | 2.90 |
| **147-154** | 5.52 | 5.07 | 4.61 | 3.94 | 3.75 | 3.20 |
| **155-163** | 6.00 | 4.89 | 4.88 | 4.30 | 4.03 | 3.25 |
| **164-173** | 5.90 | 5.44 | 4.86 | 4.29 | 3.98 | 3.38 |
| **174-185** | 6.79 | 5.68 | 5.00 | 4.38 | 3.87 | 3.69 |
| **186-201** | 6.16 | 5.89 | 5.37 | 4.75 | 4.12 | 3.58 |
| **202-220** | 6.11 | 6.34 | 5.63 | 5.09 | 4.69 | 4.40 |
| **221-247** | 6.94 | 6.47 | 5.70 | 5.00 | 4.92 | 4.42 |
| **248-292** | 7.14 | 6.49 | 5.82 | 5.37 | 5.02 | 4.40 |
| **293-399** | 7.82 | 7.09 | 6.48 | 5.79 | 5.25 | 4.56 |
| **>=400** | 8.83 | 7.45 | 7.03 | 6.51 | 6.16 | 4.59 |

**TG: triglycerides; HDL-C: high-density lipoprotein cholesterol**

**Supp.Table 17. Median statistics for the ratio of trygliserides to very low-density lipoprotein cholesterol by the cross table of non-high-density lipoprotein cholesterol and trygliserides calculated from the Turkish population (calculated for Siemens direct assay method for 420-cell strata)**

| **TG Levels (mg/dL)** | **Non-HDL-C (mg/dL)** | | | | | | | | | | | | | |
| --- | --- | --- | --- | --- | --- | --- | --- | --- | --- | --- | --- | --- | --- | --- |
|  | **<100** | **100-109** | **110-119** | **120-129** | **130-139** | **140-149** | **150-159** | **160-169** | **170-179** | **180-189** | **190-199** | **200-209** | **210-219** | **≥220** |
| **7-49** | 2.34 | 2.28 | 1.90 | 1.88 | 1.68 | 1.49 | 1.08 | 1.11 | .81 | 1.67 | .83 |  | .42 |  |
| **50-56** | 3.11 | 2.67 | 2.29 | 2.45 | 1.98 | 1.90 | 2.26 | 1.83 | 2.17 | 1.21 | 1.29 | .67 |  | .39 |
| **57-61** | 3.20 | 3.11 | 2.56 | 2.56 | 2.47 | 2.41 | 1.89 | 2.11 | 2.61 | 2.98 | 2.26 |  | 1.07 | .99 |
| **62-66** | 3.37 | 3.02 | 2.22 | 2.41 | 2.24 | 2.17 | 1.38 | 2.46 | 2.16 | 2.68 | 1.07 | 1.63 | 1.55 |  |
| **67-71** | 3.68 | 2.94 | 2.84 | 2.74 | 2.80 | 2.33 | 2.15 | 3.49 | 1.96 | 1.96 | 3.53 | 1.34 | 2.72 |  |
| **72-75** | 3.75 | 3.13 | 3.13 | 3.11 | 3.02 | 2.76 | 2.38 | 2.70 | 1.48 | 1.93 | 7.50 | 1.73 |  |  |
| **76-79** | 3.66 | 3.09 | 3.22 | 3.35 | 3.41 | 2.97 | 2.73 | 2.20 | 4.39 | 3.00 | 3.77 | 2.01 |  | .80 |
| **80-83** | 3.91 | 3.70 | 3.57 | 3.09 | 2.86 | 2.91 | 2.66 | 3.64 | 3.20 | 1.84 | 1.72 | 2.31 | 1.99 | 2.37 |
| **84-87** | 4.25 | 3.72 | 3.44 | 3.12 | 3.28 | 3.25 | 3.22 | 2.65 | 2.90 | 2.06 | 2.48 | 1.29 | 6.55 | 1.76 |
| **88-92** | 4.09 | 3.91 | 3.91 | 3.45 | 3.56 | 3.50 | 3.52 | 2.90 | 2.95 | 2.34 | 4.19 | 1.82 | 1.85 | 1.75 |
| **93-96** | 4.43 | 4.15 | 4.44 | 3.45 | 3.72 | 3.55 | 3.11 | 3.84 | 3.29 | 2.64 | 4.59 | 6.96 | 2.02 | 1.08 |
| **97-100** | 4.25 | 4.08 | 4.38 | 3.41 | 3.58 | 2.97 | 2.58 | 3.46 | 2.80 | 2.45 | 2.39 | 1.78 |  | 1.95 |
| **101-105** | 4.81 | 4.29 | 4.09 | 4.02 | 3.72 | 3.92 | 3.22 | 3.14 | 2.89 | 2.63 | 2.54 | 4.65 | 4.79 | 2.25 |
| **106-110** | 4.91 | 4.21 | 4.72 | 3.44 | 3.79 | 3.34 | 3.31 | 3.58 | 2.54 | 2.55 | 3.06 | 2.18 | 2.63 | 3.72 |
| **111-115** | 5.14 | 4.49 | 4.83 | 4.36 | 3.83 | 4.41 | 3.62 | 3.39 | 3.50 | 2.76 | 3.40 | 6.01 | 2.90 | 3.06 |
| **116-120** | 5.29 | 4.12 | 4.54 | 4.18 | 4.20 | 4.16 | 4.21 | 3.41 | 3.92 | 2.97 | 3.64 | 3.41 | 2.67 | 3.24 |
| **121-126** | 5.30 | 4.40 | 4.30 | 4.28 | 4.53 | 3.94 | 4.42 | 3.84 | 3.60 | 3.76 | 2.69 | 3.46 | 2.34 | 2.41 |
| **127-132** | 5.30 | 4.85 | 4.26 | 4.76 | 4.30 | 4.01 | 4.35 | 3.68 | 3.94 | 3.77 | 3.33 | 2.74 | 3.48 | 3.11 |
| **133-138** | 5.04 | 5.04 | 4.39 | 4.61 | 4.23 | 4.24 | 4.06 | 3.26 | 4.05 | 4.04 | 2.95 | 3.43 | 3.50 | 2.84 |
| **139-146** | 5.33 | 4.72 | 4.68 | 4.75 | 4.86 | 4.33 | 4.83 | 4.69 | 4.19 | 4.26 | 3.61 | 3.47 | 3.36 | 2.90 |
| **147-154** | 5.52 | 5.05 | 5.34 | 4.70 | 4.81 | 4.27 | 4.72 | 3.49 | 4.30 | 4.85 | 3.72 | 3.97 | 3.65 | 3.20 |
| **155-163** | 6.00 | 5.41 | 5.09 | 4.68 | 5.15 | 4.51 | 4.79 | 4.56 | 4.15 | 3.49 | 4.04 | 4.24 | 3.28 | 3.25 |
| **164-173** | 5.90 | 5.37 | 5.73 | 5.39 | 5.28 | 4.73 | 4.39 | 4.44 | 4.49 | 4.04 | 4.95 | 3.46 | 3.65 | 3.38 |
| **174-185** | 6.79 | 6.00 | 5.90 | 5.33 | 5.35 | 4.52 | 5.18 | 4.89 | 3.97 | 4.32 | 4.34 | 3.59 | 4.44 | 3.69 |
| **186-201** | 6.16 | 6.16 | 5.79 | 5.83 | 5.49 | 5.24 | 5.04 | 4.80 | 4.58 | 4.75 | 4.36 | 4.56 | 3.95 | 3.58 |
| **202-220** | 6.11 | 7.16 | 6.26 | 5.78 | 5.89 | 5.32 | 5.27 | 5.41 | 4.91 | 5.17 | 5.48 | 4.43 | 4.40 | 4.40 |
| **221-247** | 6.94 | 6.63 | 6.52 | 6.23 | 5.52 | 5.80 | 5.87 | 5.53 | 5.18 | 4.47 | 5.00 | 4.99 | 4.36 | 4.42 |
| **248-292** | 7.14 | 7.03 | 6.12 | 6.40 | 5.99 | 5.95 | 5.56 | 5.31 | 5.61 | 5.29 | 5.01 | 5.35 | 4.76 | 4.40 |
| **293-399** | 7.82 | 6.38 | 6.97 | 7.27 | 6.38 | 6.66 | 6.33 | 5.95 | 5.77 | 5.44 | 5.91 | 5.21 | 4.98 | 4.56 |
| **>=400** | 8.83 | 8.09 | 7.53 | 7.20 | 7.31 | 7.20 | 6.50 | 6.94 | 7.49 | 5.59 | 6.28 | 6.42 | 5.77 | 4.59 |

**TG: triglycerides; HDL-C: high-density lipoprotein cholesterol**

**Supp.Table 18. Median statistics for the ratio of trygliserides to very low-density lipoprotein cholesterol by the cross table of non-high-density lipoprotein cholesterol and trygliserides calculated from the Turkish population (calculated for Siemens direct assay method for 780-cell strata)**

| **TG Levels (mg/dL)** | **Non-HDL-C (mg/dL)** | | | | | | | | | | | | | | | | | | | | | | | | | |
| --- | --- | --- | --- | --- | --- | --- | --- | --- | --- | --- | --- | --- | --- | --- | --- | --- | --- | --- | --- | --- | --- | --- | --- | --- | --- | --- |
|  | **<100** | **100-104** | **105-109** | **110-114** | **115-119** | **120-124** | **125-129** | **130-134** | **135-139** | **140-144** | **145-149** | **150-154** | **155-159** | **160-164** | **165-169** | **170-174** | **175-179** | **180-184** | **185-189** | **190-194** | **195-199** | **200-204** | **205-209** | **210-214** | **215-219** | **≥220** |
| **7-49** | 2.34 | 2.31 | 1.96 | 1.85 | 1.95 | 1.85 | 1.94 | 1.67 | 1.68 | 1.55 | 1.38 | .91 | 1.13 | 1.11 |  | .30 | 2.04 |  | 1.67 | .62 | 1.04 |  |  |  | .42 |  |
| **50-56** | 3.11 | 2.67 | 2.65 | 2.08 | 2.36 | 1.94 | 2.94 | 2.17 | 1.91 | 1.72 | 1.93 | 1.27 | 2.31 | 1.83 | 1.84 | 2.17 | 2.20 | 1.21 |  | 1.29 |  | 1.77 | .67 |  |  | .39 |
| **57-61** | 3.20 | 2.62 | 3.21 | 3.17 | 2.29 | 2.58 | 2.51 | 3.05 | 2.47 | 1.88 | 2.55 | 1.81 | 1.98 | 2.11 | 3.90 | 2.69 | 2.18 | 2.98 |  | 2.26 |  |  |  | 1.21 | .93 | .99 |
| **62-66** | 3.37 | 2.96 | 3.04 | 2.23 | 2.21 | 2.42 | 2.39 | 2.24 | 2.56 | 2.44 | 2.06 | 1.34 | 1.92 | 1.48 | 2.54 | 3.88 | 2.10 | .86 | 3.54 | 1.19 | .95 | 1.21 | 1.63 | 1.68 | .99 |  |
| **67-71** | 3.68 | 2.89 | 2.97 | 2.85 | 2.59 | 2.72 | 2.76 | 2.80 | 2.79 | 2.15 | 2.51 | 2.13 | 2.41 | 3.53 | 1.66 | 1.27 | 2.17 | 1.96 | 3.90 | 3.53 |  | 1.34 |  |  | 2.72 |  |
| **72-75** | 3.75 | 3.63 | 2.91 | 2.96 | 3.27 | 3.10 | 3.26 | 3.15 | 2.31 | 2.96 | 2.61 | 2.38 | 2.37 | 2.97 | 1.59 | 1.69 | 1.48 | 1.55 | 2.51 | 7.50 |  | 1.59 | 1.77 |  |  |  |
| **76-79** | 3.66 | 3.12 | 3.07 | 3.22 | 3.15 | 3.69 | 2.93 | 3.66 | 3.39 | 2.69 | 4.94 | 2.11 | 3.57 | 1.98 | 4.11 | 7.22 | 2.91 | 3.00 |  | 2.35 | 6.00 | 2.01 |  |  |  | .80 |
| **80-83** | 3.91 | 3.86 | 3.64 | 3.45 | 3.86 | 3.42 | 2.93 | 2.74 | 3.00 | 2.56 | 3.09 | 2.58 | 2.90 | 5.05 | 3.33 | 3.20 | 2.90 | 1.64 | 2.20 | 1.32 | 1.72 | 6.84 | 1.67 | 2.64 | 1.21 | 2.37 |
| **84-87** | 4.25 | 3.74 | 3.57 | 2.96 | 3.80 | 3.11 | 3.12 | 3.29 | 3.28 | 3.58 | 2.83 | 3.20 | 3.74 | 2.83 | 2.03 | 2.77 | 4.05 | 2.06 | 2.02 | 2.48 |  | 1.29 |  | 11.62 | 1.49 | 1.76 |
| **88-92** | 4.09 | 3.87 | 3.93 | 3.79 | 3.92 | 4.05 | 3.15 | 3.58 | 3.16 | 3.26 | 4.25 | 3.62 | 3.33 | 2.90 | 2.91 | 4.06 | 2.95 | 2.78 | 1.79 | 1.99 | 6.38 | 1.97 | 1.67 | 7.86 | 1.23 | 1.75 |
| **93-96** | 4.43 | 4.10 | 4.36 | 5.05 | 3.89 | 3.80 | 3.28 | 3.10 | 4.70 | 4.05 | 3.20 | 3.20 | 2.88 | 2.88 | 3.93 | 3.84 | 3.21 | 2.82 | 1.67 | 5.00 | 4.19 | 6.96 |  | 2.02 |  | 1.08 |
| **97-100** | 4.25 | 4.00 | 4.13 | 3.75 | 5.36 | 3.25 | 3.66 | 4.17 | 3.29 | 2.77 | 3.06 | 2.33 | 2.71 | 4.04 | 2.97 | 3.72 | 2.41 | 2.51 | 2.44 | 2.80 | 2.39 | 1.78 | 2.22 |  |  | 1.95 |
| **101-105** | 4.81 | 3.85 | 4.47 | 4.16 | 4.04 | 3.94 | 4.26 | 3.85 | 3.52 | 3.81 | 4.06 | 3.55 | 2.70 | 3.41 | 3.09 | 3.64 | 2.52 | 2.63 | 2.67 | 2.54 | 2.88 | 4.65 | 4.01 | 8.36 | 2.40 | 2.25 |
| **106-110** | 4.91 | 4.41 | 3.93 | 4.56 | 5.13 | 3.42 | 3.58 | 4.02 | 3.62 | 3.39 | 3.34 | 3.34 | 3.20 | 3.57 | 3.65 | 2.04 | 2.65 | 1.88 | 2.59 | 3.29 | 2.54 | 2.62 | 1.73 | 2.63 |  | 3.72 |
| **111-115** | 5.14 | 4.38 | 4.76 | 5.16 | 4.70 | 4.73 | 3.96 | 4.13 | 3.70 | 4.48 | 4.39 | 3.80 | 3.36 | 3.39 | 3.43 | 3.65 | 3.22 | 2.83 | 2.03 | 2.46 | 3.85 | 3.00 | 23.23 | 2.83 | 2.98 | 3.06 |
| **116-120** | 5.29 | 3.90 | 4.33 | 4.46 | 4.83 | 4.47 | 4.14 | 3.70 | 4.75 | 4.45 | 3.38 | 4.58 | 3.58 | 3.49 | 3.27 | 3.92 | 4.96 | 3.13 | 2.78 | 7.81 | 3.37 | 4.00 | 2.45 | 1.94 | 3.10 | 3.24 |
| **121-126** | 5.30 | 4.49 | 4.04 | 4.41 | 4.11 | 4.19 | 4.28 | 4.42 | 4.67 | 3.51 | 4.01 | 4.42 | 4.53 | 3.21 | 4.20 | 3.75 | 3.59 | 4.33 | 3.42 | 2.16 | 2.71 | 3.46 | 3.12 | 2.13 | 4.24 | 2.41 |
| **127-132** | 5.30 | 4.87 | 4.85 | 4.03 | 4.34 | 5.02 | 4.51 | 4.54 | 3.97 | 4.08 | 4.01 | 4.35 | 4.39 | 3.68 | 3.74 | 3.67 | 5.47 | 3.66 | 4.16 | 3.48 | 3.12 | 2.95 | 2.66 | 5.20 | 1.36 | 3.11 |
| **133-138** | 5.04 | 4.59 | 5.21 | 4.09 | 4.81 | 5.10 | 4.38 | 4.28 | 4.12 | 4.76 | 3.89 | 4.03 | 4.15 | 3.58 | 3.16 | 3.94 | 4.21 | 4.05 | 4.04 | 4.14 | 2.76 | 3.43 | 5.78 | 4.02 | 3.39 | 2.84 |
| **139-146** | 5.33 | 4.67 | 4.72 | 4.66 | 4.79 | 4.77 | 4.65 | 4.66 | 5.75 | 5.82 | 4.24 | 4.83 | 4.83 | 4.26 | 4.90 | 4.21 | 4.09 | 3.74 | 5.14 | 3.45 | 3.90 | 3.74 | 3.40 | 3.54 | 3.24 | 2.90 |
| **147-154** | 5.52 | 5.65 | 4.77 | 5.21 | 5.59 | 4.50 | 4.72 | 5.03 | 4.08 | 4.13 | 4.58 | 4.49 | 4.78 | 3.68 | 3.45 | 4.30 | 4.24 | 3.02 | 5.26 | 3.75 | 3.72 | 6.68 | 3.19 | 2.84 | 5.40 | 3.20 |
| **155-163** | 6.00 | 5.26 | 5.77 | 5.31 | 4.72 | 4.52 | 4.97 | 5.61 | 4.70 | 4.53 | 4.49 | 4.78 | 4.89 | 4.30 | 4.76 | 4.74 | 3.75 | 3.55 | 3.47 | 4.04 | 4.18 | 4.45 | 3.67 | 2.72 | 5.43 | 3.25 |
| **164-173** | 5.90 | 5.93 | 4.94 | 5.22 | 6.17 | 5.44 | 5.12 | 5.49 | 5.28 | 4.66 | 4.83 | 4.90 | 4.16 | 4.61 | 4.26 | 4.83 | 3.59 | 3.81 | 4.33 | 5.31 | 4.15 | 3.62 | 3.41 | 3.35 | 4.74 | 3.38 |
| **174-185** | 6.79 | 5.69 | 6.07 | 6.07 | 5.29 | 5.30 | 5.41 | 5.52 | 4.99 | 4.45 | 4.57 | 4.60 | 5.58 | 5.07 | 4.61 | 4.09 | 3.74 | 3.74 | 4.41 | 4.47 | 3.56 | 3.22 | 3.79 | 3.42 | 6.79 | 3.69 |
| **186-201** | 6.16 | 5.77 | 6.32 | 5.05 | 6.35 | 5.89 | 5.79 | 5.54 | 5.45 | 5.44 | 5.23 | 4.96 | 5.30 | 4.79 | 4.86 | 4.68 | 4.42 | 4.63 | 5.21 | 4.42 | 4.01 | 4.41 | 4.72 | 3.82 | 5.10 | 3.58 |
| **202-220** | 6.11 | 7.54 | 6.79 | 6.47 | 6.05 | 5.56 | 5.93 | 5.86 | 6.30 | 6.42 | 4.98 | 4.95 | 5.69 | 5.48 | 5.33 | 4.92 | 4.91 | 5.34 | 4.75 | 5.01 | 5.87 | 4.67 | 4.14 | 4.93 | 4.04 | 4.40 |
| **221-247** | 6.94 | 6.24 | 6.99 | 7.37 | 6.46 | 6.22 | 6.33 | 5.41 | 5.88 | 6.21 | 5.23 | 5.89 | 5.55 | 5.83 | 4.95 | 4.99 | 5.59 | 4.48 | 4.47 | 4.92 | 5.72 | 5.16 | 4.98 | 4.05 | 4.82 | 4.42 |
| **248-292** | 7.14 | 6.80 | 7.06 | 6.88 | 5.98 | 6.37 | 6.66 | 5.82 | 6.21 | 6.60 | 5.82 | 5.41 | 5.75 | 5.20 | 5.33 | 5.40 | 5.72 | 5.27 | 5.37 | 4.99 | 5.02 | 5.52 | 5.03 | 4.82 | 3.96 | 4.40 |
| **293-399** | 7.82 | 6.37 | 6.65 | 6.47 | 7.50 | 7.47 | 7.09 | 6.30 | 6.46 | 6.51 | 6.99 | 6.52 | 6.18 | 5.92 | 6.01 | 6.03 | 5.53 | 5.51 | 5.42 | 6.13 | 5.55 | 5.26 | 4.91 | 4.95 | 5.06 | 4.56 |
| **>=400** | 8.83 | 7.52 | 9.43 | 6.37 | 8.95 | 7.50 | 7.01 | 7.25 | 7.37 | 7.89 | 7.03 | 7.14 | 6.31 | 5.99 | 7.37 | 8.17 | 7.06 | 5.99 | 5.53 | 6.55 | 6.25 | 6.29 | 6.58 | 5.94 | 5.41 | 4.59 |

**TG: triglycerides; HDL-C: high-density lipoprotein cholesterol**
